# Dual-Source Retrieval-Augmented Generation Chatbot for Women’s Health (HerCare): Design and Multimethod Evaluation Study

**DOI:** 10.2196/88549

**Published:** 2026-07-31

**Authors:** Kimia Tuz Zaman, Wordh Ul Hasan, Nova Ahmed, Juan Li

**Affiliations:** 1 Computer Science Department, North Dakota State University, 258 Quentin Burdick Building NDSU, 1320 Albrecht Boulevard, Fargo, ND, 58105, United States, 1 701-231-9662; 2 Tuskegee University, Tuskegee, AL, United States; 3 North South University, Dhaka, Dhaka Division, Bangladesh

**Keywords:** women’s health, conversational agents, large language models, retrieval-augmented generation, empathy, human-computer interaction, trust, usability

## Abstract

**Background:**

Conversational agents for women’s health often fail to meet user needs, offering either clinically sterile advice or unreliable peer anecdotes. This limitation creates a tension between the need for factual safety and emotional resonance in sensitive health contexts.

**Objective:**

We aimed to address this gap by developing and conducting a formative evaluation of HerCare, a conversational agent built on a novel dual-source retrieval-augmented generation architecture. The system integrates expert medical knowledge with peer narratives and makes the provenance of each response visible to users, enabling trust calibration through transparent source attribution.

**Methods:**

We conducted a remote, web-based single-session field study (December 2024 to January 2025; North Dakota State University Institutional Review Board Protocol #IRB0005368) with 243 completers (from 335 eligible, consenting visitors) recruited via social media (Facebook [Meta], Reddit, and Instagram [Meta]) and university mailing lists. Eligible participants self-identified as women aged 18‐45 years with English proficiency and internet access. We used a quantitative multimethod evaluation, combining standardized self-report metrics—the Chatbot Usability Questionnaire and net promoter score (NPS)—with computational linguistic analyses (VADER [Valence Aware Dictionary and Sentiment Reasoner] sentiment analysis and NRC [National Research Council] Emotion Lexicon) of 1191 conversational turns.

**Results:**

Among the 243 participants who completed the protocol, reported usability was high (Chatbot Usability Questionnaire median 78.1, IQR 65.2‐87.5; mean 75.67, SD 15.50) and advocacy was strong (NPS 60.0; 171/243, 70.4% promoters, 25/243, 10.3% detractors), though this NPS reflects completers only. Postinteraction ratings were high (all facets median 4‐5 on a 5-point scale; helpfulness, ease of use, and clarity median 5, IQR 4-5). Computational analysis revealed a consistent polarity shift from neutral to negative user queries (compound −0.18 to +0.15) to strongly positive agent responses (compound +0.55 to +0.83), with a recurring validate-then-redirect empathy pattern in which the agent acknowledges user distress before pivoting to constructive guidance.

**Conclusions:**

Among completers, the dual-source architecture was associated with high perceived empathy and trust, suggesting it can combine clinical accuracy with emotional support. These formative findings indicate the feasibility of weaving clinical sources with lived experiences toward safer, more resonant health AI and surface a candidate design pattern for future empathy-attuned systems that warrants controlled evaluation.

## Introduction

People increasingly turn to digital tools when navigating questions about fertility, contraception, periods, pregnancy, and menopause [1-5]. In these moments, they often seek not only accurate information but also language and guidance that feel respectful, reassuring, and aligned with their values [6-10]. Prior human-computer interaction (HCI) and public-health work show that women’s health experiences are shaped by emotion, culture, and trust—factors that influence whether information feels usable and safe in practice [11-17]. Yet, many systems are optimized to deliver facts quickly, filling information gaps while missing the care and empathy that could accompany the answer.

The central design challenge is not to choose between expert knowledge and lived experience, but to responsibly integrate them. Medical accuracy and safety demand grounding in vetted clinical sources; women’s health journeys are also textured by peer narratives, cultural context, and emotion that purely clinical responses cannot capture. Current systems tend to treat these domains as mutually exclusive: clinical chatbots prioritize factual correctness but strip away human resonance, while community forums provide emotional solidarity but risk misinformation [18-22]. What is missing is a principled way to weave these knowledge forms together so people do not have to choose between feeling understood and being accurately informed [14,23-25].

Retrieval-augmented generation (RAG) has emerged as a technically promising approach to grounding conversational health AI in verifiable knowledge. However, current implementations remain narrowly conceived in their knowledge sourcing. A 2025 scoping review of 67 RAG-based health studies found that 54% drew from a single knowledge source, with the large majority retrieving exclusively from clinical literature, medical guidelines, or authoritative databases [26]. Broader reviews of health care RAG systems confirm this pattern: such systems overwhelmingly retrieve from “trusted medical knowledge bases and clinical literature” [27,28], treating expert clinical content as the sole legitimate input. Community-generated health knowledge, despite representing the primary information source for many patients navigating chronic, reproductive, or stigmatized health conditions, has been systematically excluded from RAG architectures. This single-source design imposes a ceiling on empathic performance: clinically authoritative content may ground responses factually but cannot supply the experiential validation, shared language, and emotional solidarity that users of peer health communities report as distinctively valuable.

The empathy and personalization shortfall in women’s health conversational agents (CAs) is well documented. Qualitative work with pregnant users of a maternal chatbot identified limited personalization and the absence of empathetic interaction as primary weaknesses [29], and a realist synthesis of sexual and reproductive health chatbots found that systems felt less valuable when conversations were unnatural, prompting calls to prioritize authentic conversational tone and longer-term relationship building [30]. The pattern extends to perinatal and postpartum contexts: a randomized trial of a postpartum chatbot reported high satisfaction with informational content but markedly lower therapeutic-alliance scores, exposing a gap between informational adequacy and empathic presence [31]. Across these evaluations, systems optimize for accurate information while underdelivering the emotional resonance women seek in sensitive health contexts.

Taken together, this evidence defines a precise and unoccupied design space. Women’s health chatbots consistently underperform on empathy and personalization. Health RAG systems retrieve from single, clinical-only sources. Peer health knowledge is valuable but requires expert grounding to be safe. Additionally, source attribution, when architecturally enforced rather than superficially applied, measurably increases trust. No existing system has simultaneously addressed all four of these gaps at once: a single dual-source retrieval architecture that fuses vetted clinical knowledge with community peer narratives, enforces transparent source attribution at the response level, and tunes conversational tone to the user’s affective state. HerCare is designed to occupy this gap.

HerCare is a web-based CA purpose-built for women’s health support. The system combines curated clinical resources with peer narratives in a dual-source retrieval architecture and explicitly attributes which parts of a reply come from medical guidance vs community perspectives. An empathy-mapping layer tunes tone to the user’s expressed needs. While further clinical validation will be required, we use HerCare to examine whether dual-source retrieval and explicit attribution can enable safe, resonant guidance, offering groundwork for future designs in empathy-attuned health AI.

The aim of this paper is twofold: first, to describe the design and development of HerCare, a CA built on a novel dual-source RAG architecture that systematically integrates expert clinical knowledge with peer community narratives under a trust-calibration framework; and second, to report a formative evaluation of this system conducted with 243 participants in a remote, single-session field study, assessing its usability, trustworthiness, and affective dynamics through standardized self-report instruments and corpus-level computational linguistic analysis.

This work makes three contributions: (1) technical feasibility of a dual-source RAG architecture that fuses vetted clinical knowledge with peer narratives under explicit attribution; (2) a multimethod quantitative framework pairing self-report instruments with corpus-level linguistic analysis to assess affective dynamics; and (3) formative feasibility evidence characterizing the validate-then-redirect empathy pattern as a transferable design template for supportive health AI.

## Methods

### Study Design

This section is organized in two parts: system design and architecture, followed by the formative evaluation study design and procedures.

### Part 1: System Design and Development

#### HerCare System Overview and Architecture

HerCare is a web-based CA purpose-built for women’s health support. The system is built on a dual-source RAG architecture that integrates two complementary knowledge sources—vetted clinical content from the Mayo Clinic and peer narratives from Reddit women’s health communities—and explicitly attributes each piece of information to its origin. This transparent attribution is the system’s core trust-calibration mechanism: by labeling whether a response draws on community experience or medical guidance, the architecture allows users to evaluate and calibrate their trust rather than accepting outputs uncritically. An empathy-mapping layer further tunes the system’s tone to the user’s expressed emotional state before each response is generated. An overview of the complete technical software stack and deployment infrastructure is summarized in Section S6 of Multimedia Appendix 1**.**

**Figure 1.**
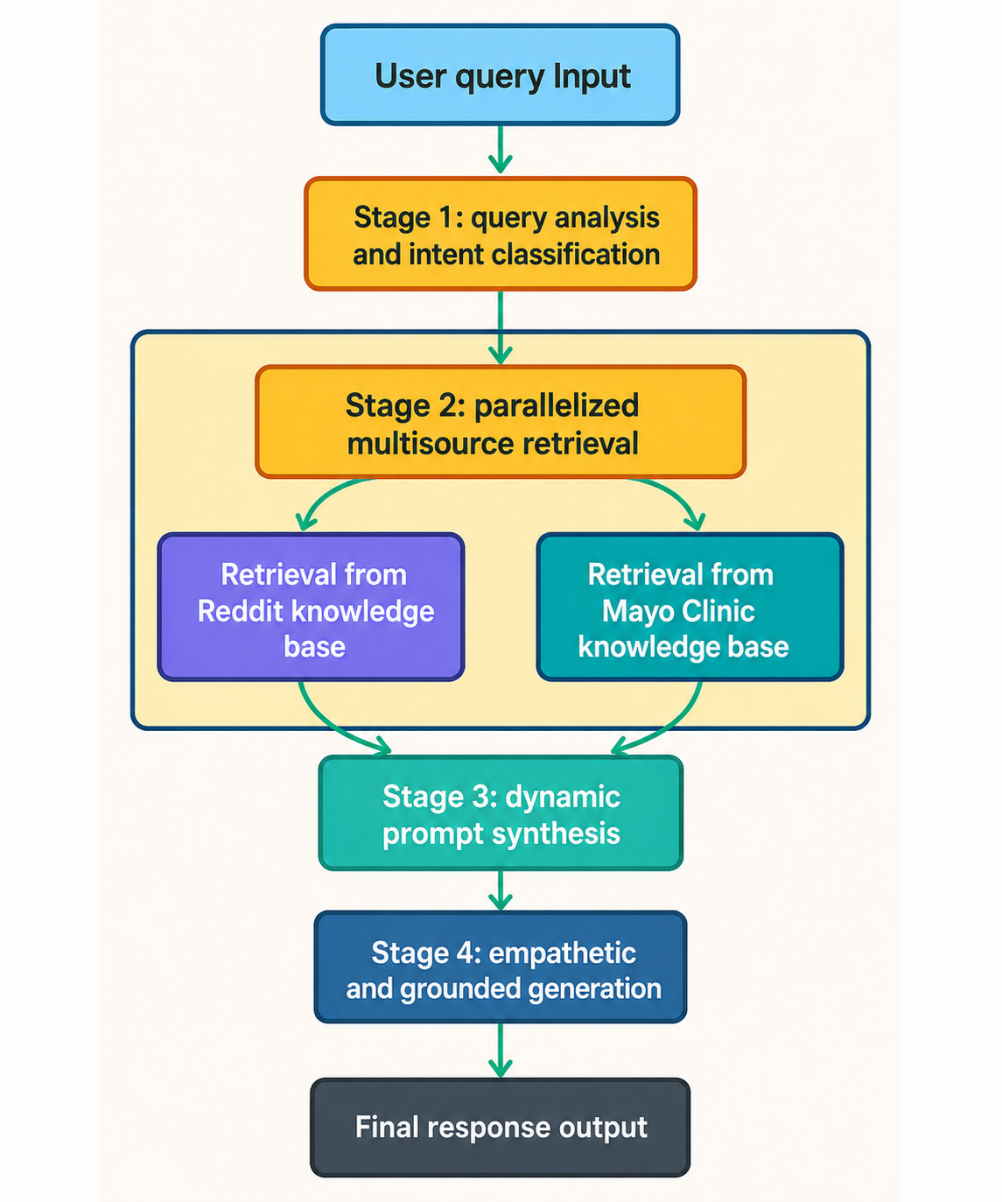
System diagram for multistaged user query. This four-stage pipeline illustrates how HerCare processes each user interaction from query analysis through parallel dual-source retrieval to empathy-informed response generation, demonstrating the architectural integration of peer and clinical knowledge sources.

The system operates through a four-stage pipeline: (1) query analysis and affective state inference, (2) parallel semantic retrieval from both vector stores, (3) dynamic prompt construction integrating the retrieved context, empathy goals, and grounding rules, and (4) response generation via GPT-4 with mandatory source attribution and a safety disclaimer. Each stage is described in the sections that follow. The complete architecture is summarized in Figure 1.

#### Knowledge Base Construction

The foundation of the system’s retrieval capability is a hybrid knowledge base composed of two distinct and purpose-built corpora. The construction of this knowledge base required the development of asymmetric data processing pipelines, where ingestion, cleaning, and chunking methodologies were specifically tailored to the unique structural and semantic characteristics of each data source. This tailored approach is critical for optimizing the quality and relevance of the information available for retrieval.

To capture the authentic voice of lived experience in women’s health, we drew our first corpus from Reddit, a social platform organized into topic-specific subreddits and shaped by pseudonymous participation. Reddit’s design encourages frank discussion of sensitive topics and produces threaded, community-moderated dialogues that are well-suited to studying support-seeking in situ. We purposively selected five active, health-relevant communities—r/WomensHealth, r/TwoXChromosomes, r/BirthControl, r/Endo, and r/PCOS (polycystic ovary syndrome)—to cover general wellness, social and personal concerns from women’s perspectives, contraceptive decision-making, and condition-specific experiences with endometriosis and polycystic ovary syndrome. Focusing on these venues allowed us to observe a broad spectrum of challenges and culturally situated narratives while remaining grounded in communities with sustained engagement and a clear fit to our research aims.

Data collection used a custom Python pipeline with authenticated API access, retrieving the top-ranked submissions per subreddit across the full subreddit history, along with all nested comments. The specific focus areas and rationales for the 5 selected online communities are detailed in Table S2 in Multimedia Appendix 1. Full collection parameters are provided in Table S1 in Multimedia Appendix 1. After deduplication and removal of unavailable items, the final corpus comprised 4995 posts and 460,317 comments across the five subreddits. Prior to vectorization, text processing, chunking, and dense embedding configurations were applied as detailed in Table S3 in Multimedia Appendix 1.

Content safety relied on a multilayer filtering strategy rather than post hoc moderation. At the corpus level, community-curated maximum-salience sampling (top-ranked threads, time_filter=‘all’) inherently prioritized content the community had collectively validated and deprioritized spam and misinformation. Thread chaining via depth-first traversal preserved within-thread corrections, ensuring that conflicting or corrected claims appeared in the same document chunk, as detailed in Section S1.3 of Multimedia Appendix 1. At the response level, the grounding and attribution instruction in the prompt explicitly prohibited presenting community anecdotes as clinical fact, and a mandatory safety disclaimer was architecturally enforced on every response. When retrieved content conflicted across sources, the prompt instructed the model to surface both perspectives with distinct attribution rather than synthesize them.

**Figure 2.**
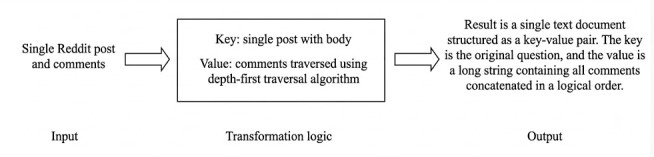
Conversational thread chaining pipeline. This transformation converts Reddit’s hierarchical comment trees into linear documents using depth-first traversal, preserving within-thread corrections and emotional context that would be lost by treating comments as isolated posts.

Reddit documents were chunked using a sentence-based strategy with sliding-window overlap to preserve emotional and semantic continuity; full parameters are in Table S3 in Multimedia Appendix 1. Before chunking, hierarchical comment trees were linearized into single documents via depth-first traversal, as illustrated in Figure 2.

The second corpus was designed to provide a foundation of factual, expert-vetted medical knowledge to ground the system’s responses and prevent the dissemination of misinformation. This corpus was constructed by systematically extracting content from the Mayo Clinic Health System’s public-facing website, a trusted source for patient-facing health information.

An automated web scraping pipeline collected content from key women’s health service pages of the Mayo Clinic Health System’s public-facing website, including birthing centers, breast cancer care, fertility, mammography, midwifery, obstetrics and gynecology, and prenatal care. The scraper was designed with strict ethical adherence, respecting the site’s robots.txt directives and implementing rate limiting to avoid server burden. Full scraping configuration, URL targets, and vector store dimensions are provided in Sections S2.1 and S2.2 and Table S4 in Multimedia Appendix 1.

#### Retrieval and Response Synthesis

With the hybrid knowledge base constructed, the next stage of the architecture is the retrieval and fusion engine. This engine is the core of the RAG system, responsible for dynamically identifying and integrating relevant information from both the community and expert corpora in real-time response to a user’s query.

When a user submits a query, it is first passed through the same embedding model used for the knowledge bases. For consistency and to ensure a meaningful comparison in the same vector space, the query is embedded using a model appropriate for the user’s likely conversational style. In this implementation, the all-MiniLM-L6-v2 model was used to vectorize the incoming query, projecting it into the same latent space as the Reddit corpus, which most closely resembles natural user language. A key design decision in this architecture is the retrieval of multiple documents rather than a single “best” match. Traditional RAG systems that retrieve only the top-ranked chunk are often brittle; if that one chunk is noisy, incomplete, or slightly off-topic, the entire generation process can be compromised, leading to a poor or irrelevant response.

To build robust context, the system retrieves 5 chunks per source (10 total per query) via cosine similarity in FAISS (Facebook AI Similarity Search). This balances GPT-4 context window constraints with informational diversity while mitigating single-chunk brittleness; full configuration is in Section S3 in Multimedia Appendix 1. Sensitivity analysis across k values remains a priority for future benchmarking.

The final stage of the architecture is the generation of the chatbot’s response. This stage is designed to synthesize the fused context from the retrieval engine into a reply that is helpful, safe, and, crucially, emotionally appropriate for the user’s situation. This is achieved through a combination of query analysis and dynamic prompt engineering, leveraging the GPT-4 large language model (LLM) for the final text generation.

#### Empathy Mapping and Prompt Engineering

To move beyond one-size-fits-all replies, the system first infers the user’s affective state from their query using the NRC (National Research Council) Emotion Lexicon, computing a length-normalized emotion profile across eight dimensions. NRC configuration details are in Section S4 in Multimedia Appendix 1.

The empathy goals and their associated response behaviors are defined in Table 1. The selected goal configures acknowledgment language, specificity and hedging, and scaffolds (eg, options lists and clinician-question checklists). The tone triad (trust, anticipation, and joy in most contexts) steers lexical choices and framing; retrieval provenance (clinical vs peer narratives) and guardrails operate independently. For auditability, each turn logs the NRC scores, dominant signal, chosen goal, and injected tone.

**Table 1.** Empathy goal.

Detected emotion (dominant signals)	Empathy goal	Response tone (triad)	Example
Distress and frustration—fear, sadness, anger	Validate and reassure	Trust, anticipation (with sadness for empathy)	“It sounds incredibly frustrating… let’s look at steps that others found empowering.”
Proactive decision-making—trust, joy, anticipation, fear	Empower and inform	Trust, joy, anticipation	“It’s great you’re being proactive… here’s what others and experts recommend.”
Ambivalence and vulnerability—sadness, disgust, trust	Normalize and support	Trust, anticipation (with sadness)	“It’s okay to feel conflicted… here’s factual info to review safely.”

The final step is dynamic prompt construction combining a persona instruction, grounding and attribution rules, an empathy tone directive, retrieved dual-source context, a task instruction, and a mandatory safety disclaimer. The full six-component prompt template is provided in Section S5 and S5.1 in Multimedia Appendix 1.

Hallucination mitigation relied on architectural grounding rather than postgeneration filtering: all responses were required to draw on retrieved Mayo Clinic content, and the prompt prohibited unsourced claims. This approach is consistent with prior work by the team and with RAG-based safety strategies documented in the literature. Formal factual consistency evaluation and independent clinical review of outputs were not conducted in this formative study and are recommended as priorities for future validation.

### Part 2: Feasibility Study Design

#### Overview

We designed the evaluation as an in-the-wild field study to understand how users would naturally interact with the HerCare agent when prompted to explore a women’s health topic of personal interest. This approach prioritizes ecological validity, allowing us to observe authentic help-seeking behaviors and capture genuine user reactions to the system’s dual-source, empathetic responses.

We chose a single-condition study design because our primary research questions were descriptive, not comparative. Our goal was to conduct an in-depth characterization of our novel architecture’s performance and affective dynamics, rather than to prove its superiority over a baseline. This approach allows for a rich understanding of the user experience with this specific type of system, forming a strong foundation for future comparative work.

The evaluation assessed three components: feasibility and acceptability via standardized self-report instruments (Chatbot Usability Questionnaire [CUQ], net promoter score [NPS], and postinteraction questionnaire [PIQ]); trustworthiness and empathy via targeted postinteraction ratings; and system affective performance via corpus-level computational linguistic analysis (latent Dirichlet allocation [LDA] topic modeling, VADER [Valence Aware Dictionary and Sentiment Reasoner] sentiment analysis, and NRC emotion profiling) of 1191 conversational turns.

This study ran for one month (December 18, 2024, to January 17, 2025). The final analytic sample comprised 243 participants who completed all study procedures, which we consider adequate for the quantitative instruments and corpus-level analyses reported here.

#### Study Population and Eligibility Criteria

Participants were required to meet the following inclusion criteria: self-identification as a woman; age between 18 and 45 years; comfort discussing intimate health topics; willingness to have chatbot interactions recorded for research analysis; ability to provide informed digital consent; access to a device with an internet connection; and English-language proficiency. Individuals were excluded if they were younger than 18 years or older than 45 years, did not identify as women, were unwilling to have interactions recorded, lacked internet access, were unable to communicate in English, or had prior involvement in the design or development of the chatbot. These criteria were established to ensure a sample with both the demographic fit and the technological access necessary for a remote, single-session web-based study.

#### Recruitment and Sample

Participants were recruited over the one-month study window via two channels: targeted posts in women’s health and wellness communities on social media platforms, and university mailing lists distributed through the research team’s professional network. To maximize reach, recruitment posts were distributed across multiple women’s health communities on Facebook, Reddit, and Instagram, and reminder posts were issued at two-week intervals throughout the recruitment window.

Recruitment materials described this study’s purpose, the voluntary and confidential nature of participation, and the remote single-session format. No compensation was offered for participation in this phase of this study. Recruitment materials are provided in Multimedia Appendix 2.

#### Data Collection Procedure

This study followed a single-session, fully remote protocol. After arriving at a secure web landing page, participants reviewed study information, provided digital informed consent via checkbox confirmation, and completed a brief demographic questionnaire collecting age range, geographic location, and religious affiliation. They were then directed to the HerCare web interface and given an open-ended naturalistic prompt—“Please interact with the chatbot about a women’s health topic of personal interest. You may ask as many questions as you like”—designed to avoid priming participants with specific scenarios and to capture authentic help-seeking behavior. Immediately following the interaction, participants completed a fixed-order postinteraction battery: the PIQ, the CUQ, and the NPS. All steps were completed in one sitting, with a median session duration of 16.9 (IQR 11.8-26.3) minutes. The 16.9-minute median duration reflects the combined time for chatbot interaction, PIQ, CUQ, and NPS completion, with no minimum interaction length required. Participants were free to end the chatbot session at any time before proceeding to the surveys. Participants who consented but did not complete the full postinteraction battery were not included in the analytic sample; the participant funnel and a comparison of completers and noncompleters are reported in the Results section.

#### Quantitative Survey Instruments

This section details the three standardized survey instruments used to collect quantitative self-reports from participants. For each instrument, we justify its selection in relation to our research questions and the system’s design goals.

We used standardized, self-report instruments to capture participants’ perceptions of the system; here we detail the CUQ [32]. CUQ is a validated, 16-item questionnaire developed for CAs. Items alternate between positively and negatively worded statements and are rated on a 5-point Likert scale (strongly disagree to strongly agree). Scores are transformed to a 0‐100 scale, facilitating interpretability and comparison with the widely used System Usability Scale.

To complement CUQ and NPS with finer-grained diagnostics, we developed a brief PIQ that targets seven facets central to our design goals of trust-calibrated, empathetic assistance: ease of use, helpfulness, clarity, speed, accuracy, trustworthiness, and empathy. The PIQ items and their response scales are presented in Table 2. The PIQ was administered immediately after the conversation to capture first-impression judgments at the point of highest salience. Each facet was measured with a single**,** targeted item rated on a 5-point Likert scale, trading breadth for low participant burden and interpretability of construct-specific responses in an in-the-wild setting. The PIQ was developed by the research team specifically for this study to target facets directly relevant to HerCare’s design goals; it has not been externally validated, which we acknowledge as a limitation of this formative evaluation.

**Table 2.** Postinteraction questionnaire: items and response scales.

Metric	Questions presented to participants	5-point Likert scale anchors
Ease of use	“How easy or difficult was it to interact with the chatbot?”	1=extremely difficult, 5=extremely easy
Helpfulness	“How helpful or unhelpful was the information provided by the chatbot?”	1=extremely unhelpful, 5=extremely helpful
Clarity	“How clear or unclear were the chatbot’s responses?”	1=extremely unclear, 5=extremely clear
Speed	“How would you rate the speed of the chatbot’s responses?”	1=extremely slow, 5=extremely fast
Accuracy	“How accurate or inaccurate did you perceive the information from the chatbot to be?”	1=extremely inaccurate, 5=extremely accurate
Trustworthiness	“How trustworthy or untrustworthy did you find the chatbot?”	1=extremely untrustworthy, 5=extremely trustworthy
Empathy	“How empathetic or unempathetic did the chatbot seem in its responses?”	1=extremely unempathetic, 5=extremely empathetic

Regarding scoring, for each metric, we summarize responses from all completers (N=243), with higher values indicating more positive assessments; item wording and anchors enable straightforward replication. Distributional normality was assessed for the CUQ total and each PIQ facet using the Shapiro-Wilk test. All measures departed significantly from normality (all *P*<.001) and were left-skewed, reflecting ceiling effects typical of high-satisfaction Likert data. We therefore report medians with IQRs as the primary descriptive statistics, retaining means and SDs only to aid comparison with prior literature.

#### Computational Analysis of Conversational Data

This section transitions from self-reported survey data to the objective, computational analysis of the raw conversational logs generated during this study. A total of 1191 user-agent conversational turns were collected from the 243 participants and subjected to analysis. This approach was chosen to complement and triangulate the subjective survey findings with objective, scalable linguistic analysis. Three specific natural language processing techniques were used to explore the nature of the interactions.

Survey responses were analyzed descriptively. As reported above, distributional normality was assessed with the Shapiro-Wilk test; because all CUQ and PIQ measures were nonnormal and left-skewed, we report medians with IQRs as the primary statistic, with means and SDs for comparison. NPS was computed as the percentage of promoters minus detractors. For the conversational corpus, the three natural language processing techniques were applied as follows: LDA first partitioned the corpus into latent topics, and VADER sentiment scores and NRC emotion profiles were then computed for each turn and aggregated by LDA-derived topic, enabling the stratified, per-topic comparisons reported in the Results section. All analyses were conducted in Python.

We used LDA to discover themes in the conversational corpus without imposing predefined categories. LDA is a generative probabilistic model for topic modeling that assumes each document—in our case, a participant’s full conversation with the agent—is a mixture of latent topics, and each topic is a probability distribution over words. Operating under a bag-of-words assumption, LDA learns both (1) topics represented by characteristic keywords and (2) per-document topic proportions, enabling us to summarize what was discussed and how strongly each theme appeared within a conversation.

We used VADER to measure sentiment polarity in both user queries and system replies. VADER is a lexicon- and rule-based tool tuned for informal text (eg, social media and conversational language). Its human-validated lexicon assigns valence scores (−4 to +4) to words, emoticons, and slang, while a rule engine adjusts for pragmatic cues such as intensifiers (“very” and “extremely”), punctuation and capitalization for emphasis, and negation (“not happy”). For any input, VADER returns proportions of positive, neutral, and negative sentiment and a normalized compound score in [−1, 1].

We used the NRC Emotion Lexicon (EmoLex) to characterize the emotional composition of dialogues at a granularity beyond simple polarity. EmoLex comprises 14k+ English terms annotated—via large-scale crowdsourcing by the NRC of Canada—with associations to eight basic emotions (anger, fear, anticipation, trust, surprise, sadness, joy, and disgust) and two sentiments (positive and negative). For each text span (eg, a user turn or system reply), we tokenize, look up lexicon matches, and aggregate counts per emotion; counts are length-normalized to yield a multidimensional emotion profile that captures the affective fingerprint of the utterance.

### Ethical Considerations

This study’s protocol was reviewed and approved by the Institutional Review Board of North Dakota State University (Protocol #IRB0005368, approved December 11, 2024, expiration December 10, 2027; Exempt, Category 3—Benign Behavioral Interventions). A protocol amendment covering additions to a subsequent phase of the broader study was approved on August 12, 2025. The research is supported by the NSF (National Science Foundation) RII (research infrastructure improvement) Track 2 FEC (focus areas competitive) grant. Participation was entirely voluntary. All prospective participants reviewed a digital information sheet detailing study procedures, potential risks, and benefits, and provided digital informed consent via checkbox confirmation on a secure online platform before any data collection. All study data were stored on encrypted, password-protected institutional servers and analyzed using deidentified datasets in which unique participant IDs replaced all personal identifiers. Research records will be retained for three years following study completion in accordance with North Dakota State University Institutional Review Board requirements, after which personally identifiable information will be permanently deleted.

The construction of the peer-support knowledge base involved data collected from public Reddit communities. Data were collected exclusively via the official Reddit API using the PRAW (Python Reddit API Wrapper), in compliance with Reddit’s terms of service. We adhered to safe harbor ethical guidelines for internet research and treated all content as sensitive given the intimate health nature of the topics discussed. A strict deidentification pipeline stripped author usernames, user flair, and specific timestamps during data ingestion. The system synthesizes retrieved narratives as aggregate, anonymous community perspectives, preventing reidentification of individual posters in the chatbot’s output. No attempt was made to contact original posters or interact with the platform beyond read-only data access.

Given the risks inherent in AI-mediated health information, the system was engineered with layered safety guardrails. Architecturally, retrieval from the Mayo Clinic corpus ensured that every response was grounded in vetted clinical content. At the prompt level, the model was explicitly prohibited from presenting community advice as medical fact and was required to attribute all information to its source. As a mandatory fail-safe, every response concluded with a safety disclaimer explicitly stating that HerCare is not a doctor and that the information provided is for educational support only and is not a substitute for professional medical advice.

## Results

### Overview

This section moves from user acceptance (CUQ, NPS, and PIQ) to corpus-level dialogue analyses, quantifying sentiment polarity and decomposing emotion profiles (VADER and NRC) by comparing user inputs with system replies. We conclude by synthesizing these layers to describe how a trust-calibrated, empathy-oriented design manifests in practice.

### Overall System Performance and User Acceptance

**Figure 3.**
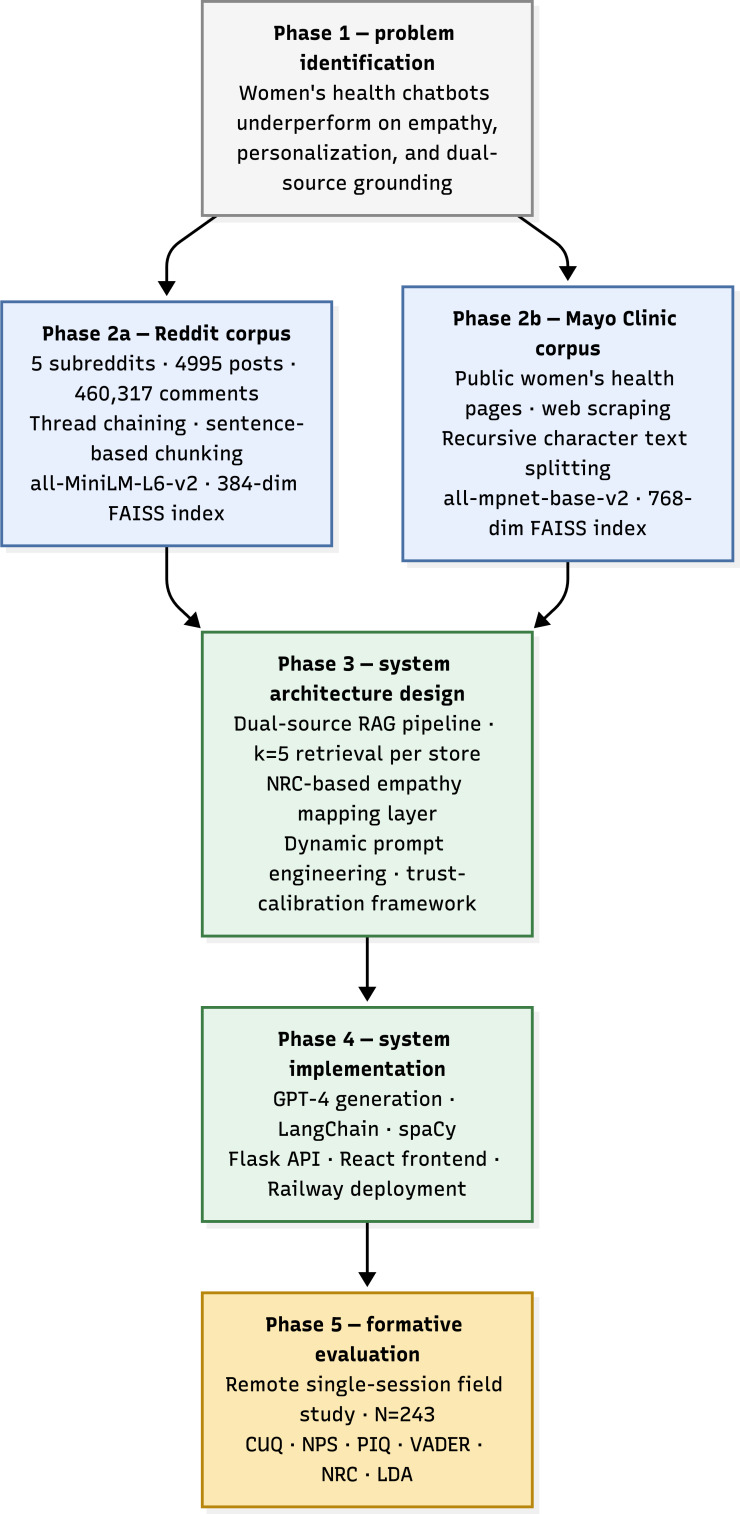
HerCare design and development process. CUQ: chatbot usability questionnaire; FAISS: Facebook AI similarity search; LDA: latent Dirichlet allocation; NPS: net promoter score; NRC: National Research Council; PIQ: postinteraction questionnaire; RAG: retrieval-augmented generation; VADER: Valence Aware Dictionary and Sentiment Reasoner.

The multistage pipeline and architectural integration of peer and clinical knowledge sources evaluated during this field study are illustrated in Figure 1. Regarding participant funnel, the public, uncompensated study link was accessed by 415 visitors during the one-month window (December 18, 2024, to January 17, 2025). The integration of this evaluation phase within the overall HerCare design and development workflow is depicted in Figure 3. Figure 3 provides an overview of the five-phase design and development process for HerCare. Phases 2a and 2b were conducted in parallel, reflecting an asymmetric corpus construction strategy in which each knowledge source received a distinct processing pipeline and embedding model optimized for its data type. Not all were participants in the intended sense: 80 did not provide eligible, informed consent (including 4 who were ineligible by age), consistent with casual exploration of a publicly advertised chatbot. Of the remaining 335 eligible, consenting visitors, 243 (72.5%) completed the full protocol (chatbot interaction, PIQ, CUQ, and NPS) and constitute the analytic sample; 92 (27.5%) did not complete the survey battery.

Regarding participant cohort, analyses draw on 243 completers (from 335 eligible, consenting visitors) in a single-session, remote study (median duration ≈16.9, IQR 11.8-26.3 min).

The sample skewed younger, predominantly US-based and Christian, with smaller Muslim and other representation (Table 3). This context informs the interpretation of acceptance and satisfaction scores.

**Table 3.** Participant demographics and overall usability metrics with comparison of completers and consented noncompleters.

Participant demographics (N=243)	Completers (n=243)	Noncompleters (n=92)	*P* value
Age (years), n (%)			.11
18‐27	117 (48.1)	53 (57.6)	
28‐37	107 (44.0)	29 (31.5)	
38‐45	19 (7.8)	10 (10.9)	
Religion, n (%)			.52
Christianity	195 (80.2)	70 (76.1)	
Islam	39 (16.0)	18 (19.6)	
Hinduism	2 (0.8)	0 (0)	
Judaism	2 (0.8)	1 (1.1)	
Other/multiple	5 (2.1)	3 (3.3)	
Geography			.052
United States	203 (83.5)	68 (73.9)	
Other	40 (16.5)	24 (26.1)	
Session duration			<.001
Median (IQR), minutes	16.9 (11.8‐26.3)	6.1 (3.6‐9.5)	
System evaluation metrics			—^a^
CUQ^b^: mean (SD)	75.67 (15.50)	—	
CUQ: median (IQR)	78.1 (65.2‐87.5)	—	
CUQ: range	37.5‐100	—	
Net promoter score	60.0	—	

a Not applicable.

b CUQ: Chatbot Usability Questionnaire.

To check for selection bias from the open recruitment, we compared completers (n=243) with consented noncompleters (n=92) on all prediscontinuation characteristics (Table 3). The groups did not differ in age (*P*=.11), religion (*P*=.52), or country (*P*=.052), but completers had longer sessions (median 16.9, IQR 11.8-26.3 vs 6.1, IQR 3.6-9.5 min; *P*<.001) and noncompleters exited at a median 40% survey progress, almost all at the questionnaire stage rather than during the chat. No noncompleter produced a complete CUQ and only one provided a PIQ or NPS response, so no analyzable partial data could be included. The shorter sessions and uniform survey-stage exit suggest many noncompleters sampled the interface out of curiosity rather than engaging in good-faith help-seeking; comparable demographics bound, without excluding, self-selection (see Limitations section).

The agent’s CUQ median was 78.1 (IQR 65.2‐87.5; mean 75.67, SD 15.50), indicating that most participants experienced the system as good to excellent in usability. The distribution was left-skewed (Shapiro-Wilk *P*<.001), with ratings concentrated at the upper end of the scale.

The seven PIQ facets clarify why top-line scores are strong. Table 4 gives details of the scores of the PIQ. The highest ratings—helpfulness**,** ease of use**,** and clarity—indicate that participants could quickly orient, obtain understandable guidance, and feel the interaction was productive. Across all facets, the median response was at the top or near-top of the scale (Table 4); accuracy, trustworthiness, and empathy showed the most spread (median 4, IQR 4‐5), suggesting these relational and correctness dimensions are judged somewhat more stringently than ease and helpfulness.

**Table 4.** Postinteraction satisfaction scores (N=243). Facets were left-skewed (Shapiro-Wilk *P*<.001); medians with IQR are the primary statistic, with means and SDs reported for comparison. Scale: 1‐5, higher is more positive.

Satisfaction metric	Median (IQR)	Average (1-5; SD)
Helpfulness	5 (4-5)	4.55 (0.65)
Ease of use	5 (4-5)	4.49 (0.80)
Clarity	5 (4-5)	4.47 (0.66)
Accuracy	4 (4-5)	4.39 (0.63)
Speed	5 (4-5)	4.33 (0.76)
Trustworthiness	4 (4-5)	4.35 (0.60)
Empathy	4 (4-5)	4.17 (0.74)

### The Conversation Polarity Shift (VADER)

**Figure 4.**
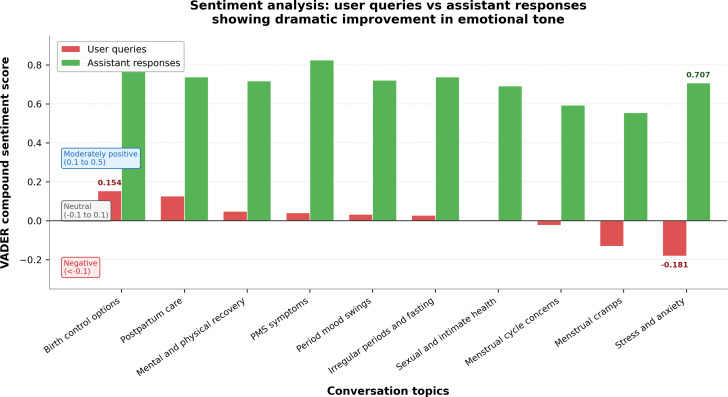
The consistent gap between neutral or negative user sentiment and strongly positive assistant sentiment is evident across all categories. Consistent with the system’s design, agent responses to distress-laden user language were uniformly supportive and forward-looking regardless of topic. PMS: premenstrual syndrome; VADER: Valence Aware Dictionary and Sentiment Reasoner.

**Figure 5.**
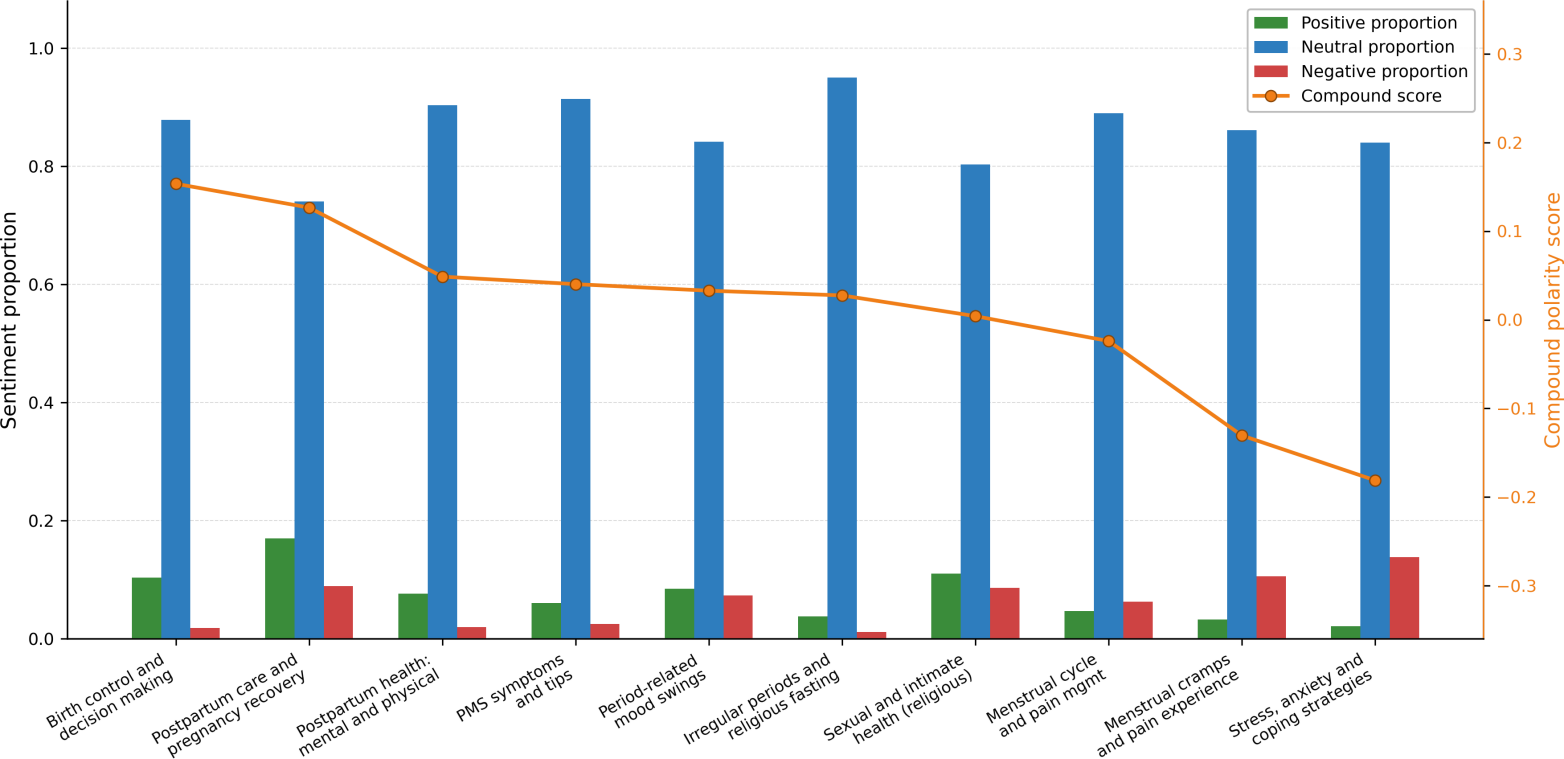
User query sentiment proportions and compound polarity scores by topic (VADER analysis). Neutral sentiment dominates across all topics (>0.74), concentrated in the informational and symptom-oriented LDA topics. Compound scores range from +0.154 (birth control options and decision-making) to −0.181 (stress, anxiety, and coping strategies), with distress-laden topics registering the most negative user language. Full per-topic data are provided in Table S1 in Multimedia Appendix 3. LDA: latent Dirichlet allocation; Mgmt: management; PMS: premenstrual syndrome; VADER: Valence Aware Dictionary and Sentiment Reasoner.

**Figure 6.**
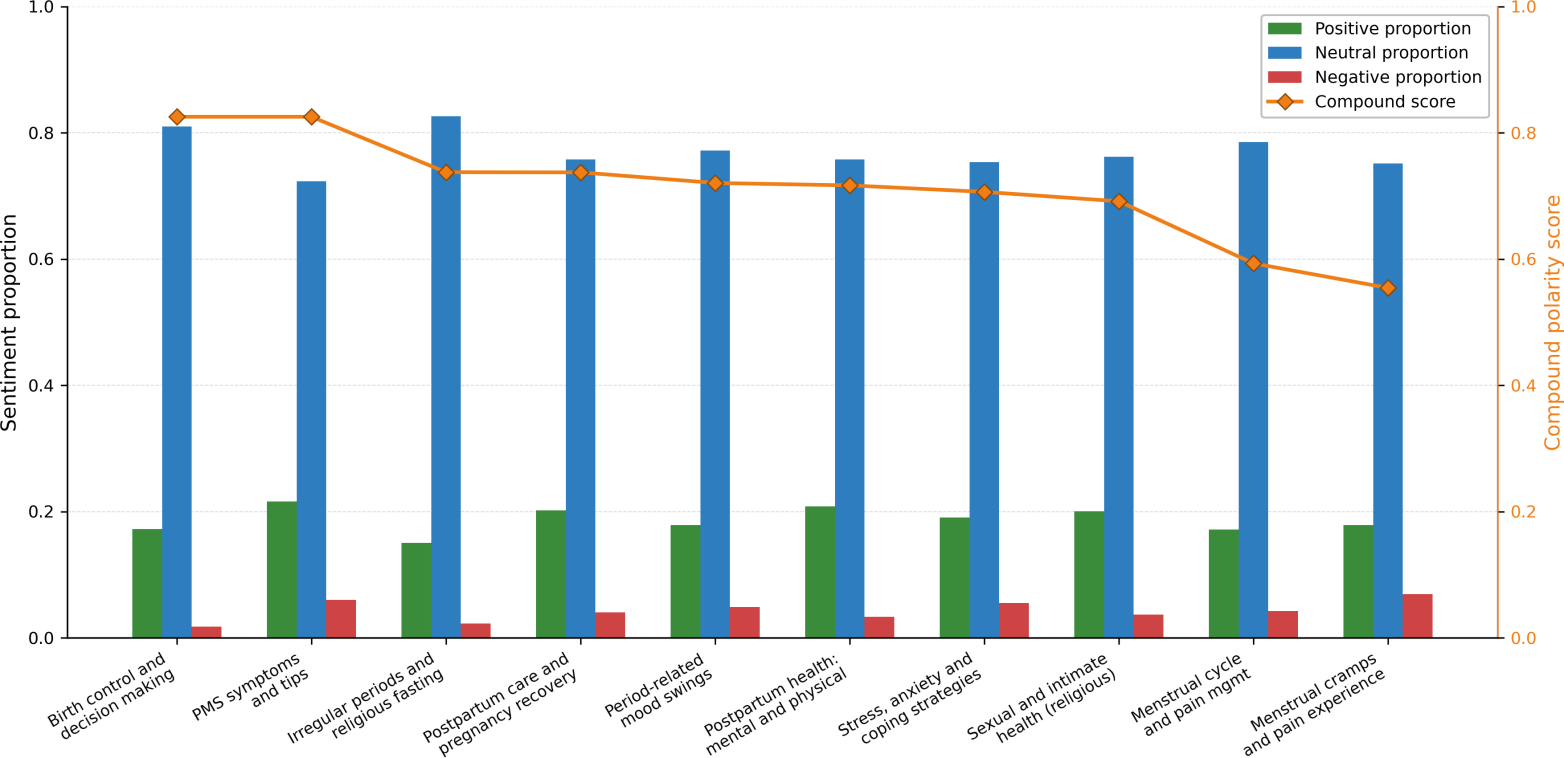
Assistant response sentiment proportions and compound polarity scores by topic (VADER analysis). In direct contrast to Figure 5, all compound scores are strongly positive (+0.555 to +0.826), and negative sentiment proportions are uniformly low (0.018‐0.069). Topics with the most negative user queries—stress, anxiety, and coping strategies, and menstrual cramps and pain experiences—still elicit robustly positive assistant responses, demonstrating that the polarity shift holds under the most affectively challenging conditions. Full per-topic data are provided in Table S2 in Multimedia Appendix 3. PMS: premenstrual syndrome.

A VADER analysis of 1191 query-response pairs shows a clear, topic-general polarity shift from user inputs to assistant replies, illustrated in Figures 4-6 below.

Overall, user turns skew neutral-to-negative, which is expected in a help-seeking setting. Across topics, the neutral component dominates (often >0.80). This neutral dominance is concentrated in the LDA-derived topics whose keyword distributions are informational and symptom-oriented (eg, menstrual cycle concerns, menstrual cramps and pain experiences, and the information-seeking facet of birth control options and decision-making), where users predominantly describe symptoms and request factual guidance; we therefore read the neutral component as fact-seeking and symptom description rather than affective flatness. Negative sentiment, by contrast, rises in the distress-laden topics. The lowest compound score appears for stress, anxiety, and coping strategies (compound −0.181), followed by menstrual cramps and pain experiences (compound −0.131), indicating language of concern, discomfort, and uncertainty. In contrast, more proactive themes register higher positivity; birth control options and decision-making show the most positive user compound (+0.154), consistent with forward-looking, choice-oriented discourse.

In marked contrast, assistant turns are consistently and strongly positive across every topic. Compound scores range from +0.555 to +0.826, with positive proportions markedly higher than in user inputs (Figure 5). Negative sentiment in replies is low across the board (0.018‐0.069), even when addressing sensitive topics such as menstrual pain or stress. This pattern indicates that responses systematically emphasize supportive, reassuring language while avoiding alarming or discouraging phrasing. Notably, topics that begin with the most negative user tone (eg, stress, anxiety, and coping strategies, and menstrual cramps and pain experiences) still elicit robustly positive assistant compounds (≥+0.554), confirming that the system maintained a positive, supportive stance even under the most affectively challenging conditions.

This systematic gap between user-query sentiment (concern) and agent-response sentiment (support) is evident topic by topic across Figures 4-6, which contrast the aggregate sentiment scores of user queries and assistant responses for each topic.

Taken together, the VADER results depict a stable user→agent positivity transition: users frequently open with neutral descriptions punctuated by negative affect in distress topics, and the agent replies with uniformly positive, supportive language across all themes. The contrast is visible topic-by-topic (Figures 4-6): the lowest user compounds remain negative while the corresponding assistant compounds fall well into the positive range, and proactive topics show positivity on both sides with a further uplift in replies. We refrain from causal claims, and we note that these analyses pool user and agent turns rather than tracking individual sessions over time; the results therefore describe an aggregate difference between user-query sentiment and agent-response sentiment, not a within-session trajectory.

### Decoding the Emotion Dialogue

**Figure 7.**
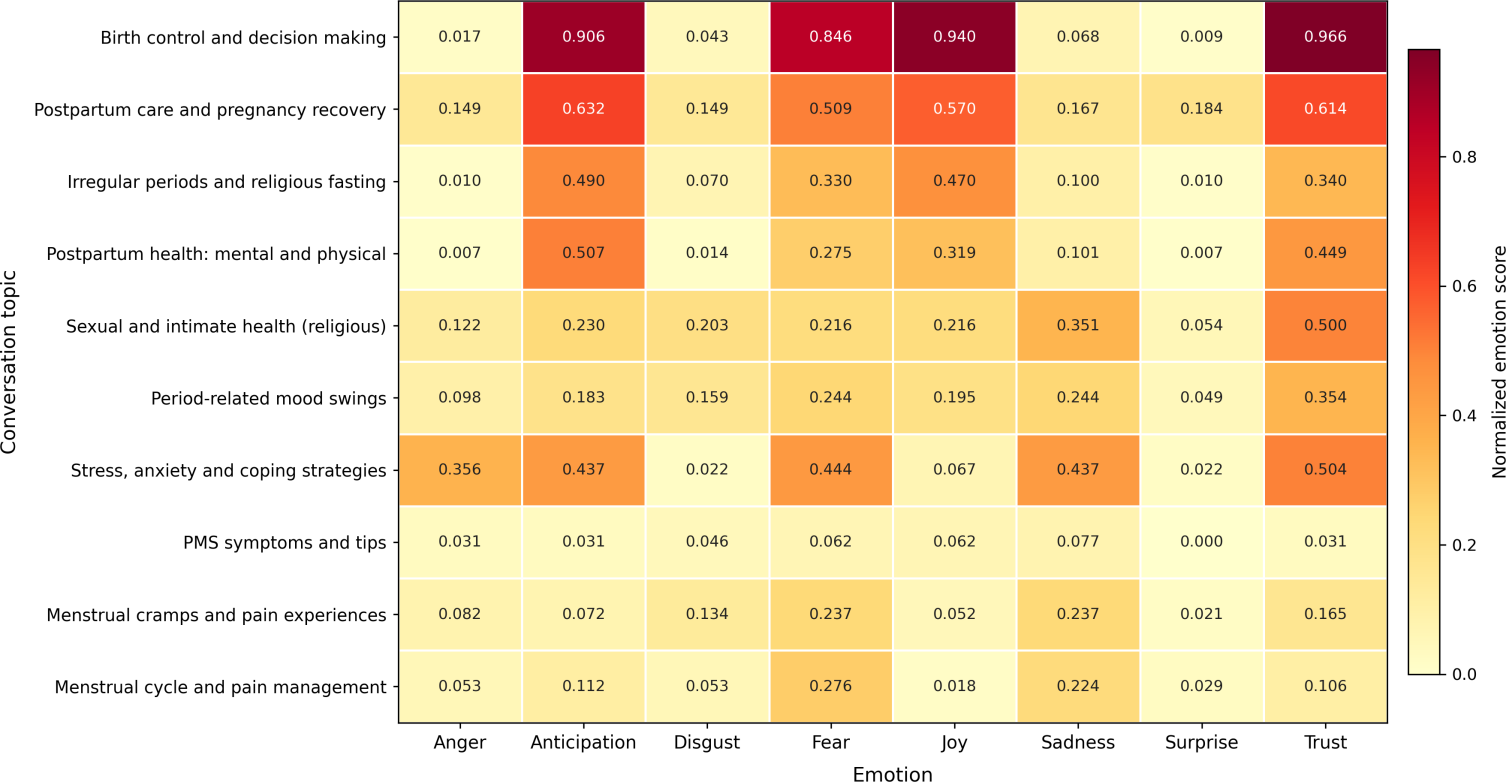
NRC emotion profiles of user queries by topic. Warmer colors indicate higher normalized emotion scores. Three distinct affective profiles are visible: a proactive profile (birth control and decision-making, top row) with elevated trust (0.966), joy (0.940), and anticipation (0.906) alongside high fear (0.846); a distress profile (stress, anxiety, and coping strategies) with elevated anger (0.356), fear (0.444), and sadness (0.437); and an ambivalence profile (sexual and intimate health in religious contexts) with elevated disgust (0.203) and sadness (0.351) alongside moderate trust (0.500). Full per-topic scores are provided in Table S3 in Multimedia Appendix 3. These three distinct profiles confirm that users brought meaningfully different emotional needs to their interactions with HerCare, providing empirical grounding for the system’s empathy-mapping design. NRC: National Research Council; PMS: premenstrual syndrome.

**Figure 8.**
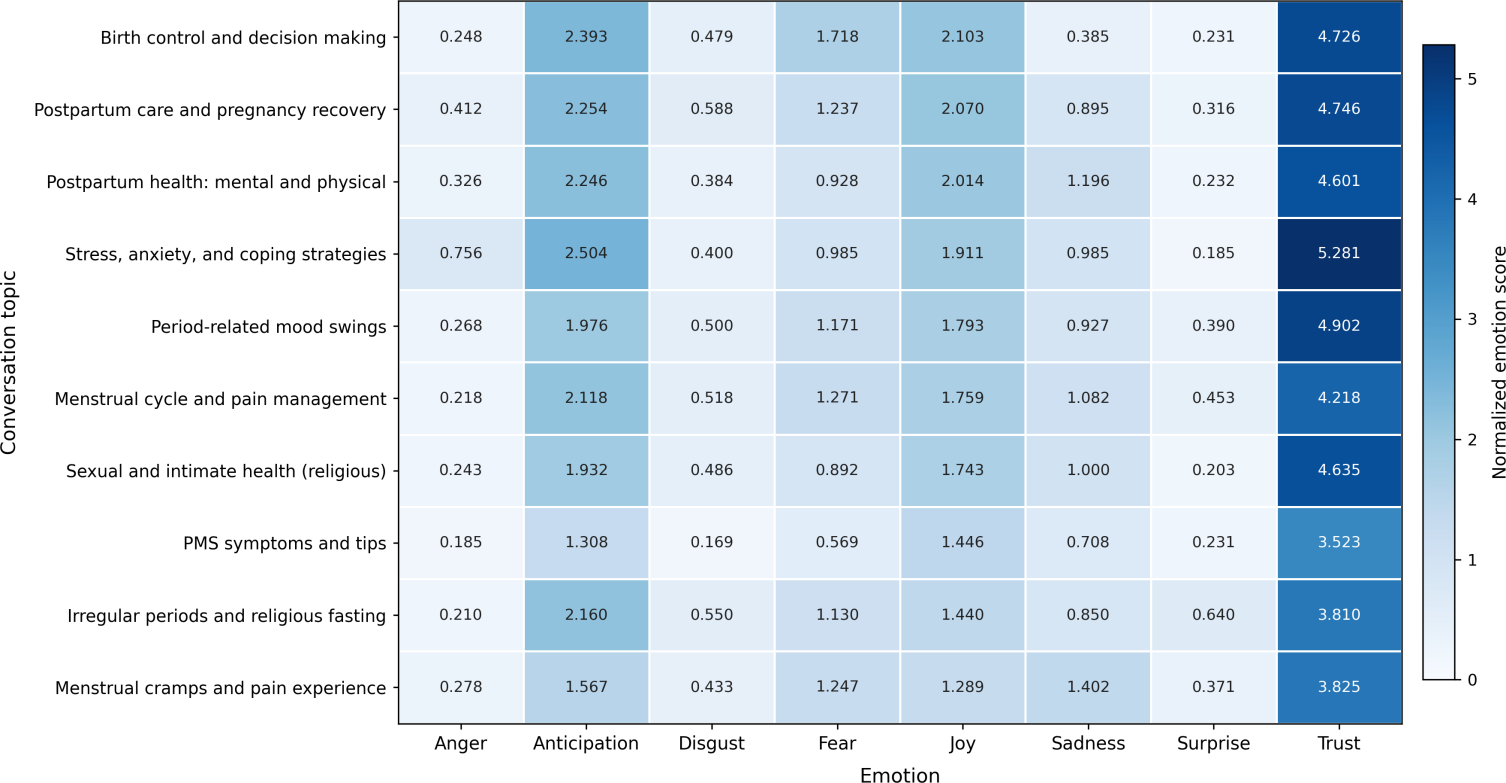
NRC emotion profiles of assistant responses by topic. The cooler blue scale reflects the higher absolute magnitude of scores relative to user queries (Figure 7). Trust dominates every row (range: 3.523‐5.281), peaking at 5.281 for stress, anxiety, and coping strategies. The validate-then-redirect pattern is visible in the menstrual cramps and pain experiences row, where sadness (1.402) is elevated alongside high trust (3.825) and anticipation (1.567), indicating that the agent briefly mirrors user distress before pivoting to constructive guidance. Full per-topic scores are provided in Table S4 in Multimedia Appendix 3. The uniform trust dominance across all topics, combined with targeted sadness elevation in distress contexts, confirms that the empathy-mapping layer successfully operationalized the validate-then-redirect strategy consistently at scale. NRC: National Research Council; PMS: premenstrual syndrome.

Using the NRC Emotion Lexicon, we profiled eight emotions—anger, anticipation, disgust, fear, joy, sadness, surprise, trust—for each turn and summarized them by topic (Figures 7 and 8). This analysis moves beyond polarity to describe the affective texture of the dialogue, indicating what users bring into the conversation and how the agent responds.

Regarding user emotional landscape, overall, user language reflects three recurring configurations. First, a proactive profile appears in birth control options and decision-making, where positive emotions co-occur at elevated levels—trust 0.966, joy 0.940, and anticipation 0.906—alongside substantial fear 0.846, consistent with high-stakes, forward-looking decision-making. Second, a distress profile characterizes stress, anxiety, and coping strategies—fear 0.444, sadness 0.437, and anger 0.356—and pain-centered topics such as menstrual cramps and menstrual cycle concerns, where fear and sadness are high, and anger is comparatively lower, indicating discomfort and uncertainty rather than frustration. Third, an ambivalence profile emerges in sexual and intimate health in religious contexts, which combines elevated sadness 0.351 and disgust 0.203 with moderate trust 0.500, suggesting emotionally conflicted help-seeking. Across topics, these distributions provide a concise map of users’ affective needs: hopeful yet apprehensive in choice-making; distressed in pain and anxiety; and conflicted when intimate concerns intersect with values and norms.

Regarding assistant emotional profile, in contrast, the agent’s replies are dominated by trust across nearly all topics, with consistently high anticipation and joy—a positivity triad that recurs throughout the corpus (Figure 8). Trust often exceeds other emotions by a wide margin, peaking at 5.281 for stress, anxiety, and coping strategies and 4.902 for period-related mood swings and symptom management. Negative emotions are present but comparatively low; importantly, sadness appears in contexts where acknowledgment is appropriate. For instance, in menstrual cramps and pain experiences, the agent shows its highest sadness 1.402, yet, this acknowledgment co-occurs with higher trust 3.825 and anticipation 1.567. This pattern is consistent with a validate-then-redirect response: the reply briefly reflects user distress (validation) before shifting emphasis toward confidence and forward-looking guidance (redirection). We observe this configuration across topics, including those with the most distressed user profiles, indicating a stable, supportive affective stance in the replies.

Taken together, the NRC results provide a granular description of how the emotional composition of conversations differs between users and the agent: users present with blends of fear, sadness, and (in places) anger or ambivalence, while replies emphasize trust and anticipation, with targeted acknowledgment where distress is salient. Figures 7 and 8 above visualize these per-topic patterns; full numerical data are provided in Tables S3 and S4 in Multimedia Appendix 3.

## Discussion

### Principal Findings

This formative study developed and evaluated HerCare, a dual-source RAG CA for women’s health. Across standardized usability instruments, postinteraction ratings, and corpus-level computational linguistic analysis, participants who completed this study rated the system highly on usability, trustworthiness, helpfulness, and empathy, consistent with good acceptability in this completer sample. Computational analysis of 1191 conversational turns confirmed that the system’s linguistic behavior aligned with its design goals: user queries skewing neutral-to-negative in affect were consistently met with strongly positive, trust-dominant responses. The designed validate-then-redirect behavior was consistently confirmed across topics, with the agent briefly acknowledging user distress before pivoting toward constructive guidance—an effective strategy enacted consistently without explicit per-topic tuning.

### Quantifying Empathy Insights From Computational Affective Analysis

The high self-reported empathy score of 4.17 of 5, together with corpus-level evidence of a systematic polarity gap and trust-dominant agent responses, is consistent with HerCare enacting its empathic design goals at the linguistic level. Our work contributes a methodological approach for looking beyond self-report scores to characterize how empathy is expressed. By triangulating user perceptions with a computational analysis of the dialogue itself, we obtain a more granular, though still descriptive, view of the system’s affective behavior.

The VADER analysis provided a clear, quantitative measure of the polarity gap between user queries and agent responses. That the agent replied to neutral or negative queries with strongly positive language confirms the system behaved as designed; the contribution here is methodological rather than an independent behavioral discovery, in that it provides a blueprint for how researchers can quantify an agent’s response sentiment relative to user input.

The NRC emotion analysis allowed for an even deeper insight, revealing the specific texture of the agent’s persona. We observed that the AI was not generically happy but appeared to act as a trust engine. Its emotional output was overwhelmingly dominated by the language of trust and anticipation, a strategic choice designed to instill confidence and hope. This observation, made possible only through computational analysis, suggests that the system’s empathetic goals were successfully translated into a measurable linguistic strategy. The most informative cross-method observation is the convergence between the agent’s elevated NRC trust and anticipation scores and participants’ independently reported empathy ratings: two methods drawing on different data sources—one a corpus-level lexical count of the agent’s output, the other a subjective postinteraction judgment by users—point in the same direction. We frame this carefully. The convergence does neither establish that the NRC profile caused the empathy ratings, nor that NRC trust is a validated proxy for perceived empathy; the two were not statistically correlated at the participant level, since NRC scores are corpus-aggregated rather than per-participant. What it does offer is triangulation: a self-report signal and a behavioral-linguistic signal that would not necessarily agree but nonetheless align, lending convergent (though not confirmatory) support to the claim that the agent’s designed affective strategy was perceptible to users. We regard establishing a per-participant link between specific emotion profiles and perceived empathy as a valuable direction for future, appropriately powered work. This multimethod quantitative approach—combining subjective user ratings with corpus-level linguistic analysis—offers a practical framework for future digital health research aiming to evaluate the affective qualities of empathetic systems.

### Validate, Then Redirect: A Design Pattern for Supportive AI

A descriptive contribution of our work is the characterization of an empathy strategy that the agent was designed to enact. The question is not just that users found the agent empathetic, but how that empathy was expressed at the linguistic level. The NRC analysis makes this concrete, confirming a consistent pattern we term validate, then redirect.

This pattern is most visible in distressing topics. For instance, in menstrual-pain discussions, the agent’s responses registered a notable level of sadness, consistent with acknowledging the user’s negative experience, alongside a substantially larger measure of trust and anticipation that shifts the response toward a constructive, hopeful frame. Rather than simple emotional mirroring, this resembles a multistep supportive-communication strategy: it signals “I hear your pain” before orienting the user toward potential solutions.

We propose validate, then redirect as a concrete and transferable design pattern for supportive AI. That this strategy was consistently operationalized by our architecture—and that users responded positively—suggests that with the right architectural foundation and prompting strategy, LLM-based systems can move beyond generic positivity toward context-aware emotional support. The articulation and refinement of such reusable design patterns, rather than their discovery as novel behaviors, is what we offer as a contribution toward more human-centered health AI.

Beyond the evaluation findings, the development of HerCare surfaced several design challenges and considerations that are broadly transferable to future empathy-attuned health AI systems. The most fundamental challenge was reconciling two epistemically different knowledge sources, peer narratives and clinical guidance, within a single response without falsely synthesizing them into a unified authoritative voice. The solution was architectural transparency: rather than merging sources into a seamless reply, the system was engineered to surface provenance explicitly in every response, allowing users to evaluate community and clinical content on their own terms. A second challenge was engineering affective responsiveness without requiring labeled emotional training data or fine-tuning: in our implementation, the NRC-based empathy-mapping layer—lexical inference over a standardized emotion lexicon combined with prompt-level goal injection—was associated with measurable empathic linguistic behavior across the corpus, suggesting this lighter-weight approach is a feasible alternative to supervised emotional modeling. Third, content quality control for Reddit data required a shift from post hoc moderation to corpus-level design relying on community voting, longitudinal engagement filters, and thread chaining to surface high-signal, collectively validated narratives rather than applying blanket content rules. Together, these design choices suggest a replicable pattern for building empathy-oriented health AI: treat knowledge provenance as a trust mechanism, use inference-based affective adaptation rather than supervised learning, and leverage community curation as a quality proxy.

### Comparison With Prior Work

Early reviews of CAs in health care showed promise but highlighted limited evidence for behavior change, small or quasi-experimental evaluations, and scarce safety reporting [33-35]. More recent syntheses continue to stress gaps in safety, evaluation quality, and appropriateness for sensitive contexts such as reproductive and intimate health [36,37]. Parallel work in women’s digital health (femtech) documents generic guidance, gaps in menopause support, and serious post-Roe privacy risks in period or fertility apps [38-44]. Studies of menstrual health apps further critique narrow personas, heteronormative defaults, and insufficient clinical grounding, despite high uptake and perceived utility [45-49]. A recent decade review consolidates these concerns, documenting Western-centric defaults and persistent underserving of Global South users, migrants, and marginalized genders, and advances a Reproductive Well-Being for All framework as a corrective lens for future systems [50]. Together, this work motivates systems that deliver accurate guidance while addressing the emotional and contextual realities of women’s health. These review gaps suggest that how information is delivered—its empathy and cultural fit—may be as consequential as correctness, motivating work on relational, tailored interactions.

Our finding that empathy ratings (4.17/5) were slightly lower than functional ratings aligns with this literature’s observation that relational qualities are judged more stringently than task performance, reinforcing the importance of investing in empathic design beyond surface-level pleasantries. Relational and empathic interaction has long been associated with better engagement, alliance, satisfaction, and even clinical outcomes [10,51,52]. HCI work on relational agents demonstrated that explicitly designed empathic behaviors (eg, responsiveness to emotion and social dialogue) can build durable alliances in health contexts [51,52]. Meta-analytic and theoretical work in health communication shows that personalization/tailoring reliably improves outcomes relative to generic messaging—when tailored to user characteristics and needs [7,8,53,54]. Public-health scholarship further distinguishes surface vs deep cultural tailoring [7]: beyond language and imagery, effective interventions embed community values, norms, and lived realities [33,34]. Within women’s intimate health, HCI studies foreground religious and cultural values shaping help-seeking and design needs, including privacy/modesty considerations and taboo navigation [6,55,56]. As a concrete Global South system example, HCI researchers co-designed a culturally appropriate AI combining community participation, professional moderation, and language sensitivity to improve fit while surfacing governance trade-offs designers must anticipate [57]. These strands collectively argue that conversational health systems should adapt to a user’s emotional state, background, and cultural context—not only to what is asked but to how it is asked. Yet, even carefully tailored clinical guidance can miss lived, context-specific concerns; online peer communities surface this missing layer.

HerCare’s dual-source architecture operationalizes what the peer support literature has long documented: that situated community knowledge complements clinical sources in ways that increase perceived relevance, particularly around taboo or culturally sensitive concerns. A large body of HCI or computer-supported cooperative work literature shows that peer spaces (eg, Reddit, WhatsApp [Meta], and Facebook groups) provide social support, reciprocity, and stigma-safe disclosure for sensitive topics including sexual abuse, postpartum depression, and broader reproductive health [58-64]. These communities often supply practical, situated knowledge that complements clinical guidance and increases perceived relevance, particularly around taboo or culturally sensitive concerns [61,63]. The design of digital safe spaces—including moderation, anonymity, and culturally appropriate norms—has been shown to be critical for women and gender minorities navigating health taboos [59,61]. This motivates architectures that can responsibly retrieve peer-support narratives alongside vetted clinical sources, while maintaining transparency about provenance and scope.

Users reported high trust in the system (4.35/5, 93% rating it trustworthy), a system that used explicit source attribution throughout. We did not, however, isolate the effect of attribution itself: participants were not asked whether attribution influenced their trust, so we cannot attribute the high trust ratings to attribution specifically. This remains an open question, of particular interest given the mixed findings on AI labeling effects in this literature. LLMs can exhibit strong performance on medical benchmarks; yet, reliability varies by task and evaluation practice—and human evaluations in health care remain uneven in design rigor [36,37,65,66]. RAG improves factuality and auditability by grounding responses in citable retrieved sources; yet, AI labeling has nuanced effects—labels can dampen perceived empathy and accuracy in some settings and often have limited impact on persuasiveness [67-69]. At the same time, disclosure and labeling present nuanced effects: AI-generated responses can make people feel heard, but explicit AI labeling can dampen perceived empathy or quality in some settings [70]; other studies find labeling has minimal impact on persuasiveness depending on context [37,70-72]. Collectively, the literature suggests health chatbots should (1) ground responses in transparent, citable sources (clinical + community where appropriate), (2) communicate uncertainty, and (3) calibrate trust through honest signaling of capabilities or limitations.

### Limitations

While our study offers preliminary evidence for the feasibility of the HerCare architecture, we acknowledge several limitations inherent to its formative nature. Our goal in this work was to characterize a novel system and establish feasibility; the following points outline the necessary next steps to build upon this foundation.

Most importantly, all outcomes are based on completers, an inherent limitation of this uncompensated, single-session design: 92 of 335 (27.5%) consenting visitors did not finish the survey battery. Completers and noncompleters did not differ significantly in measured demographics, and noncompleters overwhelmingly exited at the survey stage rather than during the interaction, but self-selection toward more engaged or favorably disposed users cannot be excluded. Usability, trustworthiness, empathy, and NPS estimates should thus be read as completer-only and may be optimistic; the NPS of 60, in particular, could be lower across all visitors. No analyzable partial data were available for noncompleters. Future deployments should add lightweight interaction measures and modest incentives to reduce survey-stage attrition.

First, this formative study provides evidence of the system’s positive reception but cannot make causal claims about its effectiveness relative to other approaches. The single-condition field study design, while maximizing ecological validity, does not include a control group. Future work should conduct a randomized controlled trial comparing our dual-source architecture against several key baselines: a single-source system using only expert clinical data, a single-source system using only peer narratives, and a generic, nonretrieval-based LLM with a similar empathetic persona. This would allow us to isolate the impact of the dual-source RAG architecture on user trust and perceived empathy.

Second, the sample’s demographic composition of the predominantly young (18-37 years), US-based (203/243, 83.5%), and Christian (195/243, 80.2%) limits generalizability in a domain where culture and religion directly shape help-seeking behavior. Women’s health experiences vary substantially across religious and cultural contexts, particularly around contraception, reproductive choices, and sexual health. The extent to which HerCare’s empathic strategies translate to non-Western, non-Christian, or older populations remains an open empirical question that future work must address through purposive sampling of underrepresented groups.

Third, both VADER and the NRC Emotion Lexicon are lexicon-based tools with known constraints in contextual sensitivity, cultural variability, and complex negation handling. They cannot detect sarcasm, irony, or culturally situated emotional expression, and their lexicons reflect primarily Western, English-language cultural associations. Findings from these analyses should be interpreted as descriptive corpus-level patterns rather than clinically precise affective measurements.

Fourth, the PIQ was developed by the research team specifically for this study and has not been externally validated. While its facets converged with validated instruments (CUQ and NPS), the lack of external validation limits the strength of construct validity claims for the trust, empathy, and accuracy facets specifically.

Fifth, as detailed in the Methods section, hallucination mitigation relied on architectural grounding and prompt-level guardrails—an approach validated in the team’s prior work rather than formal factual consistency evaluation or expert clinical review. The safety profile of HerCare’s outputs specific to women’s health topics remains to be established through independent clinical review. Additionally, this study’s window overlapped with major Western holidays, which may have elevated stress levels and introduced temporal confounds for anxiety and mood-related topics, a contextual factor that cannot be fully disentangled from the results.

Sixth, the peer corpus carries inherent demographic biases: Reddit’s user base skews younger, English-speaking, and predominantly Western, and score-based ranking may suppress minority perspectives. The five subreddits also underrepresent menopause, older women’s fertility concerns, and non-Western postpartum experiences—limitations that future studies should address through broader, more diverse corpus construction. Additionally, while transparent source attribution is a central design principle of HerCare, this study did not directly measure whether participants noticed or acted upon attribution labels; whether attribution meaningfully influenced trust calibration remains an open question for future evaluation.

Seventh, formal retrieval quality evaluation—including relevance assessment, precision metrics, and ablation of retrieval parameters—was not conducted; such benchmarking is recommended for future technical validation studies.

### Conclusions

This formative study suggests that the central tension in digital women’s health AI between clinical accuracy and emotional resonance may be addressable through architectural design. By structuring the knowledge base as two distinct, purpose-built corpora and making their provenance visible in every response, HerCare was built to let users access vetted clinical guidance and lived community experience together rather than choosing between them; establishing whether this improves outcomes relative to alternatives will require controlled comparison. The most transferable contribution is the “validate-then-redirect” pattern characterized through the corpus analysis: a candidate design template for engineering empathy in health AI that can be instantiated through prompt engineering and retrieval architecture without fine-tuning or labeled emotional training data, and that warrants validation in future comparative work. More broadly, this work suggests that health AI should be evaluated not only on accuracy but on affective alignment, whether the system’s linguistic behavior matches the emotional needs users bring to the interaction. The multimethod quantitative evaluation framework used here, combining standardized usability instruments with corpus-level computational linguistic analysis, offers a replicable methodology for assessing this alignment in future systems. Future work should pursue controlled comparative evaluation, expanded cultural and demographic diversity, formal clinical safety review, and longitudinal study of whether short-term trust and acceptance translate into sustained engagement and health behavior change.

## Supplementary material

10.2196/88549 Multimedia Appendix 1 System implementation details.

10.2196/88549 Multimedia Appendix 2 Recruitment materials and informed consent.

10.2196/88549 Multimedia Appendix 3 Supplementary computational analysis data.

## Abbreviations

CA: conversational agent
CUQ: Chatbot Usability Questionnaire
EmoLex: Emotion Lexicon
FAISS: Facebook AI Similarity Search
FEC: focus areas competitive
HCI: human-computer interaction
LDA: latent Dirichlet allocation
LLM: large language model
NPS: net promoter score
NRC: National Research Council
NSF: National Science Foundation
PCOS: polycystic ovary syndrome
PIQ: postinteraction questionnaire
PRAW: Python Reddit API Wrapper
RAG: retrieval-augmented generation
RII: research infrastructure improvement
VADER: Valence Aware Dictionary and Sentiment Reasoner
