# Supplementary material for: Dual-Source Retrieval-Augmented Generation Chatbot for Women’s Health (HerCare): Design and Multimethod Evaluation Study
Source: JMIR Form Res. 2026 Jul 31;10:e88549. doi: 10.2196/88549 (PMC13427079; doi:10.2196/88549)
Supplement: Multimedia Appendix 1 — System implementation details. [file formative-v10-e88549-s001.docx]

This appendix provides granular implementation details for the HerCare system architecture. These parameters and specifications supplement the design rationale described in the main manuscript. Researchers seeking to replicate or extend the system should refer to this appendix for the technical configuration of each pipeline component.

# S1. Reddit Corpus — Data Collection Pipeline

## Table S1. API access and collection parameters.

Data collection was conducted using the Python Reddit API Wrapper (PRAW) with authenticated OAuth2 access. The following parameters governed the collection process:

| **Parameter** | **Value** | **Rationale** |
| --- | --- | --- |
| API wrapper | PRAW (Python Reddit API Wrapper) | Official Reddit API client; compliant with terms of service |
| Authentication | OAuth2 (read-only scope) | Required for authenticated access; no write permissions used |
| Submissions per subreddit | Top 1,000 | Community-curated maximum-salience sampling |
| Time filter | time_filter="all" | Longitudinal coverage across full subreddit history |
| Ranking metric | Reddit score (upvotes − downvotes) | Community validation proxy |
| Comment depth | All nested comments (full tree) | Preserves full conversational context |
| Language filter | English-language content only | Linguistic consistency for downstream NLP |
| Metadata retained | Author handle, score, comment count, timestamp | Nonidentifying; stripped during de-identification |
| Final corpus size | 4,995 posts; 460,317 comments | After deduplication and removal of unavailable items |

## Table S2. Subreddits selected.

| **Subreddit** | **Focus Area** | **Rationale for Inclusion** |
| --- | --- | --- |
| r/WomensHealth | General women’s health | Broad coverage of wellness, symptoms, and health-seeking behavior |
| r/TwoXChromosomes | Social and personal concerns | Women’s perspectives on stigmatized and cultural health topics |
| r/BirthControl | Contraceptive decision-making | Specific focus on contraceptive options, side effects, and decisions |
| r/Endo | Endometriosis | Condition-specific community; chronic pain, diagnosis, treatment |
| r/PCOS | Polycystic ovary syndrome | Condition-specific community; hormonal, metabolic, and fertility concerns |

## S1.3 Thread Chaining Algorithm

Reddit’s hierarchical comment structure was linearized using a depth-first traversal algorithm implemented in Python. The transformation process operated as follows:

- Input: A single Reddit submission comprising a title, post body (OP), and nested comment tree.
- Traversal: A depth-first algorithm visited each comment and nested reply in sequential order, preserving parent–child relationships.
- Concatenation: All comment text was concatenated into a single continuous string, ordered by traversal sequence.
- Output: A key–value pair document where the key = post title + body (the “Question”) and the value = ordered concatenated comment string (the “Answer thread”).

This approach preserves within-thread corrections: if a community member corrected a prior post, both the original claim and the correction appear in the same document chunk, allowing the retrieval system to surface the full argumentative context.

## Table S3. Chunking and embedding — Reddit corpus.

| **Parameter** | **Value** | **Rationale** |
| --- | --- | --- |
| Chunking strategy | Sentence-based chunking | Preserves complete sentences and emotional units; avoids severing distress expressions from supportive replies |
| NLP library | spaCy within LangChain framework | Sentence boundary detection for informal conversational text |
| Chunk overlap | 50 tokens | Sliding window preserves cross-boundary narrative continuity |
| Embedding model | all-MiniLM-L6-v2 (Sentence-Transformers) | Optimized for informal, conversational text; favorable accuracy–efficiency balance |
| Embedding dimensions | 384-dimensional vectors | Compact representation suitable for large-volume conversational corpus |
| Vector index | FAISS (Facebook AI Similarity Search) | Efficient approximate nearest-neighbor search at scale |

# S2. Mayo Clinic Corpus — Data Collection Pipeline

## S2.1. Web scraping configuration.

Expert medical content was collected from the Mayo Clinic Health System’s public-facing website using an automated scraping pipeline developed in Python. The following ethical and technical parameters governed the process:

| **Parameter** | **Value** | **Rationale** |
| --- | --- | --- |
| Scraping scope | Public women’s health service pages (see S2.2) | Targets relevant clinical content |
| robots.txt compliance | Full compliance; scraper respected all crawl directives | Ethical web scraping |
| Rate limiting | Implemented to avoid server burden | Responsible data collection |
| User-agent header | Appropriately identified research crawler | Transparent collection |
| HTML parsing library | BeautifulSoup (Python) | Robust HTML parsing |
| Content extracted | Main body text only (paragraphs, headings, lists) | Signal extraction |
| Content excluded | Navigation menus, headers, footers, sidebars, JavaScript, boilerplate | Noise reduction |

## S2.2 Pages Scraped

The scraper targeted the following women’s health service sections of the Mayo Clinic Health System website:

- Birthing Centers
- Breast Cancer Care
- Fertility
- Mammography
- Midwifery
- OB-GYN
- Prenatal Care

## Table S4. Chunking and embedding — Mayo Clinic corpus.

| **Parameter** | **Value** | **Rationale** |
| --- | --- | --- |
| Chunking strategy | Recursive Character Text Splitter | Preserves document’s inherent semantic structure (paragraphs, sections, list items) |
| Separator hierarchy | Double newline → single newline → space | Aligns chunks with semantic units; superior to fixed-length splitting for structured documents |
| Embedding model | all-mpnet-base-v2 (Sentence-Transformers) | Higher representational accuracy for formal, well-structured clinical text |
| Embedding dimensions | 768-dimensional vectors | Higher-dimensional representation captures nuanced clinical semantics |
| Vector index | FAISS (separate from Reddit index) | Maintained as an independent vector store to preserve source distinction |

# S3. Retrieval Configuration

| **Parameter** | **Value** | **Rationale** |
| --- | --- | --- |
| Query embedding model | all-MiniLM-L6-v2 | Projects user query into same latent space as Reddit corpus, which most closely resembles natural user language |
| Chunks retrieved per store | k = 5 | Balances context window constraints, informational diversity, and generative coherence; consistent with RAG heuristic practice for sensitive domains |
| Total chunks per query | 10 (5 Reddit + 5 Mayo Clinic) | Ensures dual-source representation in every response |
| Similarity metric | Cosine similarity (FAISS default) | Standard for semantic retrieval in dense vector space |
| Retrieval architecture | Parallel dual-store retrieval | Both stores queried independently and simultaneously; results fused in prompt context |

*Note: k=5 was selected through contextual reasoning optimizing for GPT-4 context window management and informational diversity rather than empirical grid search. Sensitivity analysis across values of k is recommended in future technical benchmarking studies.*

# S4. Empathy Mapping — NRC Emotion Lexicon Configuration

| **Parameter** | **Value** |
| --- | --- |
| Lexicon | NRC Emotion Lexicon (EmoLex), National Research Council Canada |
| Lexicon size | 14,000+ English terms |
| Emotions scored | Trust, Anticipation, Joy, Fear, Sadness, Anger, Disgust, Surprise |
| Normalization | Length-normalized per-emotion counts (token-level aggregation) |
| Dominant signal selection | Highest normalized score determines empathy goal |
| Auditability logging | Per-turn log of NRC scores, dominant signal, empathy goal, and injected tone triad |

The three empathy goals and their corresponding tone triads are described in Table 1 of the main manuscript.

# S5. Response Generation — GPT-4 Configuration

The following table details the full configuration of the GPT-4 response generation stage, including API parameters, generation settings, and prompt-level design decisions. These parameters were held constant across all participant interactions.

| **Parameter** | **Value** | **Rationale** |
| --- | --- | --- |
| Language model | GPT-4 (OpenAI API) | State-of-the-art instruction-following LLM; strong performance on nuanced, multi-constraint prompts |
| API version | gpt-4 (stable release, OpenAI Chat Completions API) | Stable production version used throughout the study window (December 2024 – January 2025) |
| Temperature | 0.7 | Balances response creativity and consistency; avoids deterministic repetition while maintaining factual grounding |
| Max tokens | 1,000 | Sufficient for comprehensive, multi-part responses incorporating both community and clinical context without truncation |
| Top-p (nucleus sampling) | 1.0 (default) | No additional top-p filtering applied; temperature setting provided adequate output diversity |
| Frequency penalty | 0.0 (default) | No frequency penalty applied; responses grounded in retrieved content rather than free generation |
| Presence penalty | 0.0 (default) | No presence penalty applied |
| Prompt structure | Six-component dynamic prompt | Persona → Grounding/Attribution → Dynamic Empathy Tone → Context Injection → Task Instruction → Safety Disclaimer |
| Attribution enforcement | Mandatory source labeling in prompt instructions | Strict rule: community content labeled as peer perspective; Mayo Clinic content labeled as expert guidance |
| Safety disclaimer | Appended to every response; architecturally enforced via prompt | States AI is not a doctor; not a substitute for professional medical advice |
| Post-generation filtering | None applied | Safety enforced at prompt level; no output filtering layer used in this formative study |
| Persona | Compassionate, non-clinical women’s health support assistant | Warm, supportive tone; explicitly non-diagnostic |
| Medical advice prohibition | Explicit prompt instruction | Model instructed: “you are not a doctor and you cannot give medical advice” |

*Note: Hallucination mitigation in this formative study relied on architectural grounding (mandatory retrieval from vetted Mayo Clinic content) and prompt-level prohibition of unsourced claims. Formal factual consistency evaluation or expert clinical review of outputs was not conducted. Assessment of hallucination rates and formal safety evaluation are recommended priorities for future validation studies.*

## S5.1 Full Six-Component Prompt Template

The following is the complete prompt template assembled dynamically for each user query. Bracketed placeholders ({EMPATHY_GOAL}, {TONE_DESCRIPTION}, {COMMUNITY_CONTEXT}, {CLINICAL_CONTEXT}, {USER_QUESTION}) are populated at runtime from the empathy-mapping output and retrieved chunks. This template was held constant across all participant interactions during the study window (December 2024–January 2025).

**Component 1 — Persona Instruction:**

You are a compassionate, knowledgeable, and safe women’s health support assistant. You are designed to provide supportive information by combining insights from peer communities and expert medical sources. You are not a doctor and you cannot give medical advice.

**Component 2 — Grounding and Attribution Instruction:**

You will answer the user’s question by synthesizing information from two sources provided below: ‘Community Context’ from Reddit discussions and ‘Expert Context’ from the Mayo Clinic. You MUST clearly attribute which information comes from which source. For example, use phrases like ‘Many in the community have shared...’ or ‘According to the Mayo Clinic...’. Never present community advice as medical fact. This is a strict rule.

**Component 3 — Empathy Tone Instruction (Dynamic, populated at runtime):**

The user is expressing {DETECTED_EMOTIONS}. Your primary empathy goal is to {EMPATHY_GOAL}. {TONE_DESCRIPTION}. Example (Proactive Decision-Making profile): The user is expressing Trust, Joy, Anticipation and Fear. Your primary goal is to Empower and Inform. Acknowledge their proactiveness, but frame your response in an encouraging and positive tone. Share hopeful peer experiences alongside clinical facts.

**Component 4 — Context Injection:**

### Community Context (from Reddit): {COMMUNITY_CONTEXT} ### Expert Context (from Mayo Clinic): {CLINICAL_CONTEXT}

**Component 5 — Task Instruction:**

User’s Question: “{USER_QUESTION}” Based on all the above instructions and context, provide a helpful and empathetic response to the user’s question. Always attribute information to its source.

**Component 6 — Safety Disclaimer Instruction:**

Conclude your entire response with the following disclaimer, separated by a line: ‘Please remember, this information is for support and educational purposes only and is not a substitute for professional medical advice. Always consult with a qualified healthcare provider for any health concerns or before making any decisions related to your health.’

When community narratives and clinical content conflict, Components 2 and 3 operate in concert: the Attribution Instruction prohibits presenting peer anecdote as clinical fact, while the mandatory source-labeling language (“Many in the community have shared...” versus “According to the Mayo Clinic...”) makes divergence visible to the user rather than concealing it within a synthesized response. Component 6 appends a safety disclaimer to every response as a final architectural safeguard.

# S6. System Stack Summary

| **Component** | **Technology** | **Role** |
| --- | --- | --- |
| Reddit data collection | Python + PRAW | API-based corpus construction |
| Web scraping | Python + BeautifulSoup | Mayo Clinic content extraction |
| NLP pipeline | spaCy + LangChain | Sentence chunking and embedding orchestration |
| Reddit embedding | all-MiniLM-L6-v2 | 384-dim conversational text embeddings |
| Mayo Clinic embedding | all-mpnet-base-v2 | 768-dim clinical text embeddings |
| Vector storage | FAISS (two separate indices) | Approximate nearest-neighbor retrieval |
| Response generation | GPT-4 (OpenAI API) | Final response synthesis |
| Backend API | Flask (Python) | Server-side application logic |
| Frontend | React | Web-based chat interface |
| Deployment | Railway | Cloud hosting platform |
| Empathy mapping | NRC Emotion Lexicon | Affective state inference from user queries |
