# Supplementary material for: Dual-Source Retrieval-Augmented Generation Chatbot for Women’s Health (HerCare): Design and Multimethod Evaluation Study
Source: JMIR Form Res. 2026 Jul 31;10:e88549. doi: 10.2196/88549 (PMC13427079; doi:10.2196/88549)
Supplement: Multimedia Appendix 2 — Recruitment materials and informed consent. [file formative-v10-e88549-s002.docx]

This appendix contains the recruitment and consent materials used in the HerCare formative evaluation study, organized in four sections: (S1) recruitment overview and channel strategy, (S2) verbatim recruitment post, (S3) informed consent summary, and (S4) full informed consent form as administered to participants.

# S1. Recruitment Overview

## Table S1. Recruitment strategy summary.

| **Parameter** | **Detail** |
| --- | --- |
| Recruitment window | December 18, 2024 – January 17, 2025 (one month) |
| Target population | Women aged 18–45, English-speaking, comfortable with intimate health topics |
| Channels used | Social media (Facebook, Reddit, Instagram); university mailing lists; researcher network |
| Reminder cadence | Reminder posts issued at two-week intervals throughout recruitment window |
| Compensation | None for Phase 1 (this study). Phase 2 interview participants received $20 online gift card. |
| Study link | https://tinyurl.com/HerCare-Survey |
| Chatbot link | https://hercare.pythonanywhere.com/ |
| Contact | kimia.zaman@ndsu.edu |
| Consent method | Digital checkbox confirmation on secure online platform |
| IRB approval | Protocol #IRB0005368, NDSU, December 11, 2024 (Exempt, Category 3) |

## S1.1 Channel Distribution Detail

Recruitment notices were distributed through the following channels to maximize reach:

- **Facebook Groups:** Women’s health, reproductive health, and maternal wellness communities.
- **Reddit Communities:** Posted in r/WomensHealth, r/TwoXChromosomes, r/BirthControl, r/Endo, and r/PCOS.
- **Instagram:** Study graphic and post text shared via research team accounts and relevant community spaces.
- **University Mailing Lists:** Distributed via researcher network at North Dakota State University.
- **Researcher Network:** Direct email outreach through professional contacts in women’s health and AI research.

## S1.3 Eligibility Criteria

- Women aged 18–45
- Comfortable discussing intimate health topics
- Internet access and a compatible device
- English-language proficiency
- Willing to provide digital informed consent

# S2. Recruitment Post — Verbatim Text

The following is the verbatim text of the recruitment post distributed across Facebook, Reddit, Instagram, and university mailing lists. Formatting, hashtags, and links are reproduced as distributed.

| **Participate in a Study to Improve Women’s Health Support!**  Are you passionate about shaping the future of women’s health technology? We’re looking for women aged 18–45 to join a study evaluating a culturally sensitive chatbot designed to assist with intimate health concerns like menstrual health, reproductive health, and more.  **What You’ll Do:**   - **Interact** with the chatbot to complete simple, scenario-based tasks. - **Share feedback** through short surveys (Phase 1). - **Optional:** Join a follow-up interview (Phase 2) for in-depth feedback.   **What’s in It for You:**   - **Make an impact** on the development of an empathetic health tool. - Participants selected for Phase 2 will receive a $20 online gift card upon completing the interview!   **Who Can Participate:**   - Women aged 18–45 - Comfortable discussing intimate health topics - Have internet access and a device to interact with the chatbot   **How to Join:**  Learn more and sign up: <https://tinyurl.com/HerCare-Survey>  Try the chatbot directly: <https://hercare.pythonanywhere.com/>  Your participation is confidential and will help improve health tools for women worldwide.  Questions? Contact: [kimia.zaman@ndsu.edu](mailto:kimia.zaman@ndsu.edu)  Thank you for being part of this important research!  #WomensHealth #HealthTech #AIForGood #MenstrualHealth #ReproductiveHealth #ResearchStudy #Empowerment #TechForWomen #HealthSupport |
| --- |

*Note: Per NDSU IRB reviewer feedback, the recruitment graphic was revised prior to deployment to clearly distinguish Phase 1 (uncompensated) from Phase 2 ($20 gift card), ensuring participants understood the two-phase structure.*

# S3. Informed Consent Summary

Digital informed consent was obtained from all participants via checkbox confirmation on a secure online platform prior to any data collection. Participants were informed of the following key elements:

- Study purpose: evaluating the usability, empathy, and trustworthiness of an AI women’s health support chatbot
- Data collected: anonymized chatbot interaction logs, survey responses, and demographic information
- Data handling: anonymized using unique participant IDs; stored on encrypted institutional servers; retained 3 years post-study per NDSU IRB requirements
- Voluntary participation: participants could withdraw at any time without consequence
- Confidentiality: only aggregate, de-identified results reported in publications
- IRB oversight: NDSU IRB Protocol #IRB0005368, Exempt Category 3, approved December 11, 2024

The full consent form as administered to participants is reproduced in Section S4 below.

# S4. Full Informed Consent Form — As Administered

*The following is the complete text of the informed consent form presented to all participants prior to enrollment. The form was administered digitally via a secure online platform. Participants indicated consent by checkbox selection.*

| **Computer Science Department**  North Dakota State University  Fargo, ND 58108-6050  **Evaluating the Usability and Cultural Sensitivity of an Empathetic Chatbot for Women’s Health Support**  **This study is being conducted by:**  Principal Investigator: Dr. Juan Li (PI) — j.li@ndsu.edu  Researcher: Kimia Tuz Zaman (Doctoral GRA) — kimia.zaman@ndsu.edu  **Key Information about this study:**  This study aims to evaluate a chatbot designed to assist women with intimate health concerns such as menstrual health, reproductive health, and mental well-being. The chatbot is intended to provide culturally sensitive, accurate, and empathetic responses.  **What You Need to Know:**   - **Eligibility:** Women aged 18–45, comfortable discussing intimate health topics, and with access to proper logistics and some basic idea on using chatbots. - **Time Commitment:** Phase 1: 10–20 minutes. Phase 2: 1–1.5 hours (if selected). - **Risks:** Minimal risk, primarily related to potential discomfort in discussing sensitive topics. Measures are in place to protect your privacy and confidentiality. - **Benefits:** Gain insights into managing health topics and contribute to the development of a culturally sensitive health tool. - **Compensation:** Participants completing Phase 2 will receive a $20 online gift card. - **Confidentiality:** Your information will be kept confidential and anonymized, and only the research team will have access to it.   **Why am I being asked to take part in this study?**  You are being asked to participate in this study because we are seeking insights from women who may have health questions related to intimate topics such as menstrual and reproductive health. Your experiences and feedback can help us understand how well the chatbot supports individuals like you and how we can improve its accuracy, empathy, and cultural sensitivity. By sharing your thoughts and interactions, you can contribute to creating a supportive and private tool for women’s health.  **What will I be asked to do?**  **Phase 1:** You will interact with a chatbot to complete scenario-based tasks. While the scenario and goal provide a starting point, you are welcome to be creative in how you engage with the chatbot. Your chatbot interaction history will be recorded for the study, anonymized, and analyzed to evaluate usability, effectiveness, and cultural sensitivity. After completing each task, you will answer a brief survey about your experience.  **Phase 2:** At Phase 1, you will be asked if you are interested in participating in a follow-up interview for Phase 2. From the pool of interested participants, we will select a diverse group for this phase. Selected participants will engage in a one-on-one virtual interview lasting 1 to 1.5 hours covering trust, privacy, empathy, and cultural sensitivity. Participants selected for Phase 2 will receive a $20 online gift card as compensation for their time.  **Where is the study going to take place, and how long will it take?**  The study will take place entirely online:   - Phase 1: Complete chatbot tasks and surveys at your convenience (10–20 minutes). - Phase 2: Interviews will be conducted via secure video conferencing software (1–1.5 hours).   **What are the risks and discomforts?**  It is not possible to identify all potential risks in research. However, you might experience potential emotional discomfort when discussing sensitive topics and privacy concerns related to collected data. We have implemented strong safeguards to minimize these risks, including anonymization, encryption, and secure data storage. If new findings develop during the course of the research which may change your willingness to participate, we will tell you about these findings.  **What are the expected benefits of this research?**   - **Individual Benefits:** Learn more about managing intimate health topics and influence the development of a supportive health tool. - **Societal Benefits:** Contribute to the creation of a culturally sensitive chatbot to support women’s health globally.   **Do I have to take part in this study?**  No. Participation is completely voluntary. You may withdraw at any time without penalty or loss of benefits.  **Who will have access to my information?**  If you agree to participate in Phase 2, your email address will be collected and securely stored to contact you for scheduling interviews and providing compensation. Only authorized research team members will have access to this information, which will be deleted within 30 days after Phase 2 is completed.  Audio and video recordings from Phase 2 interviews will be securely stored and used only for transcription and analysis by the research team. These recordings will not include your name or other direct identifiers in the transcription process. Your email and recordings will not be shared outside the research team without your explicit consent. Data will be anonymized and stored securely. Results will be presented in aggregate form without identifying participants.  **How will my information be used?**   - **Email Address:** If you opt into Phase 2, your email address will be used solely for contacting you to schedule interviews and to provide the $20 online gift card compensation. - **Chat Histories and Survey Responses:** These will be analyzed to evaluate the chatbot’s usability, effectiveness, and cultural sensitivity. - **Audio/Video Recordings:** If you participate in Phase 2, recordings of your interview will be transcribed and analyzed by the research team. These recordings will not include your name or other direct identifiers in the transcription process. - **Demographic Data:** This will help ensure diversity in our analysis and understand how the chatbot performs for various cultural and educational backgrounds.   All identifiable information will be stored securely, separated from your responses, and anonymized during analysis. Once the study is completed, identifiable information will be deleted within 30 days, and only de-identified data will be retained for future research and reporting purposes.  **Can my participation in the study end early?**   - **Voluntary Withdrawal:** You may choose to stop participating at any time, for any reason, without penalty or loss of benefits. - **Eligibility Issues:** If it is determined that you do not meet the inclusion criteria after enrollment, your participation may be discontinued. - **Study Requirements:** If you are unable to complete the tasks or provide the necessary data, your participation may be ended.   If your participation ends early, any data collected up to that point will be securely retained unless you request its removal. There will be no impact on your relationship with the research team or any affiliated organization.  **Will I receive any compensation for participating in the study?**  Participants who complete Phase 2 will receive a $20 online gift card.  **What if I have questions?**  Before you decide whether you’d like to participate in this study, please ask any questions that come to mind now. Later, if you have questions about the study, you can contact Dr. Juan Li at j.li@ndsu.edu, or Kimia Tuz Zaman at kimia.zaman@ndsu.edu.  **What are my rights as a research participant?**  You have rights as a research participant. All research with human participants is reviewed by a committee called the Institutional Review Board (IRB) which works to protect your rights and welfare. If you have questions about your rights, an unresolved question, a concern or complaint about this research you may contact the Research Integrity & Compliance office at 701.231.8908 or via email (ndsu.irb@ndsu.edu).  **After reviewing the consent form, are you willing to participate in this study?**  Please note that your participation is entirely voluntary, and you can withdraw at any time without any consequences.   - Yes, I am willing to participate in this study - No, I do not wish to participate in this study   **Documentation of Informed Consent**  You are freely making a decision whether to be in this research study. Signing this form means that you understand all the conditions and agree to participate in the study. Please select the following terms.   - I have read and understood this consent form - I have had my questions answered - I have decided to be in this study   **I understand all the terms and agree to participate**  Your Printed Name: ______________________________  Date: ______________________________  Sign / Initial: ______________________________  **Email Address (Optional)**  Please provide your email if you are willing to participate (if selected) in the second phase of this study. Phase 2 involves a one-on-one interview with a research team member to discuss your experience with the chatbot. The goal is to understand your emotional responses, comfort level, privacy concerns, and the chatbot’s cultural sensitivity. If you participate in this phase, you will receive a $20 digital gift card as compensation for your time. The interview will be recorded, transcribed, and analyzed, but your recordings will be anonymized, with no personal identifiers included in the transcription process.  Email: ______________________________ |
| --- |
