# Supplementary material for: Dual-Source Retrieval-Augmented Generation Chatbot for Women’s Health (HerCare): Design and Multimethod Evaluation Study
Source: JMIR Form Res. 2026 Jul 31;10:e88549. doi: 10.2196/88549 (PMC13427079; doi:10.2196/88549)
Supplement: Multimedia Appendix 3 — Supplementary computational analysis data. [file formative-v10-e88549-s003.docx]

This appendix provides full per-topic numerical data for the four computational analyses visualized in Figures 4–8 of the main manuscript. Tables S1 and S2 contain VADER sentiment scores for user queries and assistant responses respectively. Tables S3 and S4 contain NRC Emotion Lexicon scores for user queries and assistant responses respectively.

**Contents**

S1. VADER Sentiment Analysis — User Queries (Figure 5 data)

S2. VADER Sentiment Analysis — Assistant Responses (Figure 6 data)

S3. NRC Emotion Analysis — User Queries (Figure 7 data)

S4. NRC Emotion Analysis — Assistant Responses (Figure 8 data)

# S1. VADER Sentiment Analysis — User Queries

Table S1 provides the full per-topic VADER sentiment scores for user queries, corresponding to Figure 5 in the main manuscript. Topics are ordered by compound score descending. Neutral sentiment dominates across all topics (range: 0.740–0.950), consistent with factual help-seeking language. Compound scores range from +0.154 (Birth Control Options & Decision-Making) to −0.181 (Stress, Anxiety & Coping Strategies).

| **Dominant Topic** | **Negative Proportion** | **Neutral Proportion** | **Positive Proportion** | **Compound Score** | **Conversation Count** |
| --- | --- | --- | --- | --- | --- |
| Birth Control Options & Decision-Making | 0.0180 | 0.8786 | 0.1034 | 0.1536 | 117 |
| Postpartum Care & Pregnancy Recovery | 0.0896 | 0.7402 | 0.1702 | 0.1267 | 114 |
| Postpartum Health: Mental & Physical Recovery | 0.0201 | 0.9033 | 0.0766 | 0.0488 | 138 |
| Premenstrual Syndrome (PMS) Symptoms & Tips | 0.0254 | 0.9139 | 0.0607 | 0.0403 | 65 |
| Period-Related Mood Swings & Symptom Management | 0.0734 | 0.8417 | 0.0849 | 0.0329 | 82 |
| Irregular Periods & Religious Fasting | 0.0118 | 0.9502 | 0.0380 | 0.0276 | 100 |
| Sexual & Intimate Health in Religious Contexts | 0.0861 | 0.8033 | 0.1106 | 0.0042 | 74 |
| Menstrual Cycle Concerns & Pain Management | 0.0632 | 0.8901 | 0.0467 | -0.0239 | 170 |
| Menstrual Cramps & Pain Experiences | 0.1058 | 0.8612 | 0.0330 | -0.1305 | 97 |
| Stress, Anxiety, & Coping Strategies | 0.1381 | 0.8404 | 0.0215 | -0.1811 | 135 |

*Table S1. VADER sentiment analysis of user queries by dominant conversation topic (N = 1,191 conversational turns). Negative proportion = proportion of words with negative valence; Neutral proportion = proportion of words with neutral valence; Positive proportion = proportion of words with positive valence; Compound score = normalized composite polarity score ranging from −1 (most negative) to +1 (most positive); Conversation count = number of conversations assigned to each topic by LDA.*

# S2. VADER Sentiment Analysis — Assistant Responses

Table S2 provides the full per-topic VADER sentiment scores for assistant responses, corresponding to Figure 6 in the main manuscript. Topics are ordered by compound score descending. All compound scores are strongly positive (range: +0.555 to +0.826) and negative proportions are consistently low (range: 0.018–0.069), confirming a uniform supportive register across all conversation themes regardless of topic distress level.

| **Dominant Topic** | **Negative Proportion** | **Neutral Proportion** | **Positive Proportion** | **Compound Score** | **Conversation Count** |
| --- | --- | --- | --- | --- | --- |
| Birth Control Options & Decision-Making | 0.0177 | 0.8100 | 0.1724 | 0.8256 | 117 |
| Premenstrual Syndrome (PMS) Symptoms & Tips | 0.0604 | 0.7232 | 0.2164 | 0.8256 | 65 |
| Irregular Periods & Religious Fasting | 0.0231 | 0.8261 | 0.1508 | 0.7378 | 100 |
| Postpartum Care & Pregnancy Recovery | 0.0403 | 0.7580 | 0.2017 | 0.7375 | 114 |
| Period-Related Mood Swings & Symptom Management | 0.0490 | 0.7723 | 0.1786 | 0.7207 | 82 |
| Postpartum Health: Mental & Physical Recovery | 0.0336 | 0.7581 | 0.2084 | 0.7169 | 138 |
| Stress, Anxiety, & Coping Strategies | 0.0555 | 0.7536 | 0.1908 | 0.7066 | 135 |
| Sexual & Intimate Health in Religious Contexts | 0.0371 | 0.7623 | 0.2007 | 0.6917 | 74 |
| Menstrual Cycle Concerns & Pain Management | 0.0426 | 0.7856 | 0.1718 | 0.5933 | 170 |
| Menstrual Cramps & Pain Experiences | 0.0693 | 0.7518 | 0.1789 | 0.5545 | 97 |

*Table S2. VADER sentiment analysis of assistant responses by dominant conversation topic. Column definitions are identical to Table S1. The contrast with Table S1 is stark: all compound scores are strongly positive and negative proportions are uniformly low, demonstrating a systematic polarity shift from user queries to assistant responses across every topic.*

# S3. NRC Emotion Analysis — User Queries

Table S3 provides the full per-topic NRC Emotion Lexicon scores for user queries, corresponding to Figure 7 in the main manuscript. Scores represent length-normalized mean emotion counts per conversation turn. Three recurring affective configurations are identifiable: a proactive profile in Birth Control & Decision-Making (elevated Trust, Joy, Anticipation, Fear); a distress profile in Stress, Anxiety & Coping Strategies and pain-centered topics (elevated Anger, Fear, Sadness); and an ambivalence profile in Sexual & Intimate Health in Religious Contexts (elevated Disgust, Sadness alongside moderate Trust).

| **Topic** | **Anger** | **Anticipation** | **Disgust** | **Fear** | **Joy** | **Sadness** | **Surprise** | **Trust** |
| --- | --- | --- | --- | --- | --- | --- | --- | --- |
| Birth Control Options & Decision-Making | 0.017 | 0.906 | 0.043 | 0.846 | 0.940 | 0.068 | 0.009 | 0.966 |
| Postpartum Care & Pregnancy Recovery | 0.149 | 0.632 | 0.149 | 0.509 | 0.570 | 0.167 | 0.184 | 0.614 |
| Irregular Periods & Religious Fasting | 0.010 | 0.490 | 0.070 | 0.330 | 0.470 | 0.100 | 0.010 | 0.340 |
| Postpartum Health: Mental & Physical Recovery | 0.007 | 0.507 | 0.014 | 0.275 | 0.319 | 0.101 | 0.007 | 0.449 |
| Sexual & Intimate Health in Religious Contexts | 0.122 | 0.230 | 0.203 | 0.216 | 0.216 | 0.351 | 0.054 | 0.500 |
| Period-Related Mood Swings & Symptom Mgmt | 0.098 | 0.183 | 0.159 | 0.244 | 0.195 | 0.244 | 0.049 | 0.354 |
| Stress, Anxiety, & Coping Strategies | 0.356 | 0.437 | 0.022 | 0.444 | 0.067 | 0.437 | 0.022 | 0.504 |
| Premenstrual Syndrome (PMS) Symptoms & Tips | 0.031 | 0.031 | 0.046 | 0.062 | 0.062 | 0.077 | 0.000 | 0.031 |
| Menstrual Cramps & Pain Experiences | 0.082 | 0.072 | 0.134 | 0.237 | 0.052 | 0.237 | 0.021 | 0.165 |
| Menstrual Cycle Concerns & Pain Management | 0.053 | 0.112 | 0.053 | 0.276 | 0.018 | 0.224 | 0.029 | 0.106 |

*Table S3. NRC Emotion Lexicon analysis of user queries (mean length-normalized emotion scores per topic). All eight basic emotions are scored: Anger, Anticipation, Disgust, Fear, Joy, Sadness, Surprise, Trust. Scores represent the mean frequency of words associated with each emotion per conversational turn, normalized by turn length. Higher values indicate stronger presence of that emotion in user language for that topic.*

# S4. NRC Emotion Analysis — Assistant Responses

Table S4 provides the full per-topic NRC Emotion Lexicon scores for assistant responses, corresponding to Figure 8 in the main manuscript. Note that absolute score magnitudes are substantially higher than user query scores in Table S3, reflecting the longer and richer nature of assistant responses. Trust dominates across all ten topics (range: 3.523–5.281). The Validate-then-Redirect pattern described in the main text is most visible in the Menstrual Cramps & Pain Experiences row, where Sadness (1.402) is elevated alongside high Trust (3.825) and Anticipation (1.567).

| **Topic** | **Anger** | **Anticipation** | **Disgust** | **Fear** | **Joy** | **Sadness** | **Surprise** | **Trust** |
| --- | --- | --- | --- | --- | --- | --- | --- | --- |
| Birth Control Options & Decision-Making | 0.248 | 2.393 | 0.479 | 1.718 | 2.103 | 0.385 | 0.231 | 4.726 |
| Postpartum Care & Pregnancy Recovery | 0.412 | 2.254 | 0.588 | 1.237 | 2.070 | 0.895 | 0.316 | 4.746 |
| Postpartum Health: Mental & Physical Recovery | 0.326 | 2.246 | 0.384 | 0.928 | 2.014 | 1.196 | 0.232 | 4.601 |
| Stress, Anxiety, & Coping Strategies | 0.756 | 2.504 | 0.400 | 0.985 | 1.911 | 0.985 | 0.185 | 5.281 |
| Period-Related Mood Swings & Symptom Mgmt | 0.268 | 1.976 | 0.500 | 1.171 | 1.793 | 0.927 | 0.390 | 4.902 |
| Menstrual Cycle Concerns & Pain Management | 0.218 | 2.118 | 0.518 | 1.271 | 1.759 | 1.082 | 0.453 | 4.218 |
| Sexual & Intimate Health in Religious Contexts | 0.243 | 1.932 | 0.486 | 0.892 | 1.743 | 1.000 | 0.203 | 4.635 |
| Premenstrual Syndrome (PMS) Symptoms & Tips | 0.185 | 1.308 | 0.169 | 0.569 | 1.446 | 0.708 | 0.231 | 3.523 |
| Irregular Periods & Religious Fasting | 0.210 | 2.160 | 0.550 | 1.130 | 1.440 | 0.850 | 0.640 | 3.810 |
| Menstrual Cramps & Pain Experiences | 0.278 | 1.567 | 0.433 | 1.247 | 1.289 | 1.402 | 0.371 | 3.825 |

*Table S4. NRC Emotion Lexicon analysis of assistant responses (mean length-normalized emotion scores per topic). Column definitions are identical to Table S3. The consistently dominant Trust scores across all rows, combined with targeted Sadness elevation in distress-laden topics, confirm that the system’s empathy-mapping layer successfully enforced a stable, forward-oriented affective stance while briefly acknowledging user distress where appropriate.*
